# The Physiological Link between Metabolic Rate Depression and Tau Phosphorylation in Mammalian Hibernation

**DOI:** 10.1371/journal.pone.0014530

**Published:** 2011-01-18

**Authors:** Jens T. Stieler, Torsten Bullmann, Franziska Kohl, Øivind Tøien, Martina K. Brückner, Wolfgang Härtig, Brian M. Barnes, Thomas Arendt

**Affiliations:** 1 Department of Molecular and Cellular Mechanisms of Neurodegeneration, Paul Flechsig Institute of Brain Research, University of Leipzig, Leipzig, Germany; 2 Institute of Medical Microbiology and Hygiene, University Medical Centre of the Johannes Gutenberg-University Mainz, Mainz, Germany; 3 Institute of Arctic Biology, University of Alaska Fairbanks, Fairbanks, Alaska, United States of America; 4 Department of Pathophysiology of Neuroglia, Paul Flechsig Institute of Brain Research, University of Leipzig, Leipzig, Germany; National Institutes of Health, United States of America

## Abstract

Abnormal phosphorylation and aggregation of tau protein are hallmarks of a variety of neurological disorders, including Alzheimer's disease (AD). Increased tau phosphorylation is assumed to represent an early event in pathogenesis and a pivotal aspect for aggregation and formation of neurofibrillary tangles. However, the regulation of tau phosphorylation *in vivo* and the causes for its increased stage of phosphorylation in AD are still not well understood, a fact that is primarily based on the lack of adequate animal models. Recently we described the reversible formation of highly phosphorylated tau protein in hibernating European ground squirrels. Hence, mammalian hibernation represents a model system very well suited to study molecular mechanisms of both tau phosphorylation and dephosphorylation under *in vivo* physiological conditions. Here, we analysed the extent and kinetics of hibernation-state dependent tau phosphorylation in various brain regions of three species of hibernating mammals: arctic ground squirrels, Syrian hamsters and black bears. Overall, tau protein was highly phosphorylated in torpor states and phosphorylation levels decreased after arousal in all species. Differences between brain regions, hibernation-states and phosphosites were observed with respect to degree and kinetics of tau phosphorylation. Furthermore, we tested the phosphate net turnover of tau protein to analyse potential alterations in kinase and/or phosphatase activities during hibernation. Our results demonstrate that the hibernation-state dependent phosphorylation of tau protein is specifically regulated but involves, in addition, passive, temperature driven regulatory mechanisms. By determining the activity-state profile for key enzymes of tau phosphorylation we could identify kinases potentially involved in the differentially regulated, reversible tau phosphorylation that occurs during hibernation. We show that in black bears hibernation is associated with conformational changes of highly phosphorylated tau protein that are typically related to neuropathological alterations. The particular hibernation characteristics of black bears with a continuous torpor period and an only slightly decreased body temperature, therefore, potentially reflects the limitations of this adaptive reaction pattern and, thus, might indicate a transitional state of a physiological process.

## Introduction

Tau is an axonally located, microtubule-associated protein that is encoded by a single gene and predominantly expressed in neurons [1]. Tau mRNA transcripts can be spliced alternatively, and the expression of tau-isoforms is developmentally regulated and varies between species [2]–[4]. In the adult human brain tau is expressed in six isoforms that differ with respect to the number of N-terminal inserts and microtubule binding repeats [2]. Due to binding to tubulin, tau promotes assembly and stability of microtubules [5], [6]. The tau-microtubule interaction is a dynamic process that plays a pivotal role in structural remodelling of the cytoskeleton during neuronal and synaptic plasticity [7]. The binding capacity of tau to microtubules is regulated at different levels. The expression of four instead of three microtubule binding repeats results in tau-isoforms that differ in affinity to microtubules [8]. In addition, protein modification by phosphorylation represents a very rapid mechanism to regulate the binding capacity of tau. Phosphorylation of tau is a physiological process and elevated phospho-degrees give rise to a decreased microtubule binding [9]–. In early ontogenesis tau protein is highly phosphorylated which promotes a flexible microtubule network for neuronal plasticity and synaptogenesis during development [10], [12].

**Figure 1:**
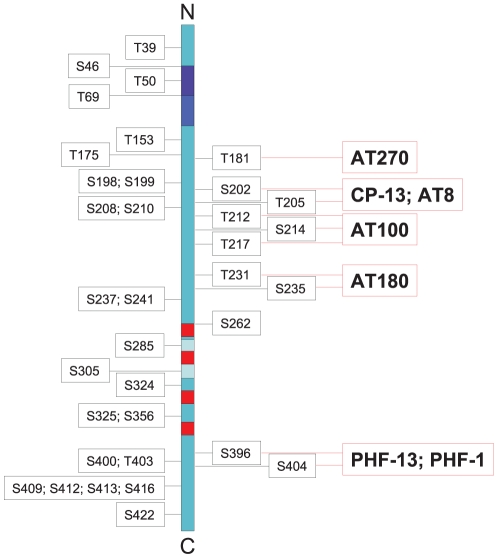
Model of tau protein with potential phosphosites. The analysed tau phosphosites are indicated on the right designating the applied antibodies.

A variety of neurodegenerative disorders is characterised by the formation of intracellular deposits of phosphorylated tau protein aggregated into paired helical filaments (PHF) [13]. For example, neurofibrillary tangles (NFT) consisting of PHF-tau represent a major hallmark of Alzheimer's disease (AD), the most prominent type of so-called “tauopathies”. Aggregated tau protein differs from normal tau by its high degree of phosphorylation, its conformation as well as its solubility [9], [14]. Still, little is known about functional links between degree of phosphorylation and aggregation of tau protein. Tau phosphorylation can induce conformational changes that subsequently modulate its propensity for self-aggregation [15], [16]. Moreover, phosphorylation of tau can promote self-assembly and filament formation, at least under *in vitro* conditions [17]–[19]. On the other hand, phosphorylation may also lessen PHF-tau assembly [20]. Thus, depending on the particular phospho-site, tau protein aggregation can either be promoted or impaired [21]–[23]. In the human tau protein more than 30 phosphorylation sites have been identified as being involved in both physiological and pathological processes (Figure 1). It had been hypothesised [24]–[26] that tau phosphorylation in AD may initially represent a physiological reaction with a protective function that in the course of pathogenesis eventually turns into a pathological result. However, the lack of appropriate *in vivo* models of PHF-like tau phosphorylation so far impedes a proof of this concept.

We have demonstrated a PHF-like phosphorylation of tau in hibernating mammals [24], [27], [28], a finding that recently has been replicated by other groups [29]. In the state of torpor with decreased metabolism and body temperature, brains of hibernating animals show an intensely elevated PHF-like pattern of tau phosphorylation which is fully reversed when animals return to the normal state after arousal [24], [27]. Furthermore, torpor in hibernating animals shows significant analogies to the pathophysiological condition of AD with respect to an altered synaptic connectivity [24], [30]–[33], the types of neurons affected [27], and the impairment of cognitive function [34].

Therefore, mammalian hibernation represents a model system very well suited to analyse conditions and mechanisms physiologically associated with increased tau phosphorylation and altered synaptic connectivity. In addition, the hibernation model helps to identify potential differences between tau hyperphosphorylation in torpor and AD, thereby contributing to our understanding of the significance of tau phosphorylation for neurodegeneration.

In the present study we analysed the reversible tau phosphorylation in arctic ground squirrels (*Spermophilus parryii*), Syrian hamsters (*Mesocricetus auratus*) and black bears (*Ursus americanus*). These species were selected since they differ with respect to their hibernation characteristics as well as their degree of body temperature change during torpor. With a body temperature of about 0°C arctic ground squirrels show the most extreme reduction followed by the Syrian hamsters where body temperature is lowered to about 5°C during hibernation. Black bears, however, show only a slight temperature change during hibernation with a body temperature of no lower than 30°C. In arctic ground squirrels and Syrian hamsters hibernation is characterised by regular torpor intervals interrupted by brief arousal episodes, where metabolic rate and body temperature of animals returns to normal levels. On the contrary, black bears do not show such regular arousal episodes.

Therefore we were able to determine that tau phosphorylation is a general, hibernation-related phenomenon and that different natures of hibernation and/or divergent hibernation conditions do not result in a different tau phosphorylation signature.

The three species were compared using a panel of phosphorylation-dependent tau antibodies that was applied to different brain regions. To analyse whether hibernation-state related tau phosphorylation is a specifically regulated process or passively driven by a brain temperature-dependent shift of kinase- and phosphatase activities, we assessed the phosphate net turnover of tau protein in different hibernation-states. Moreover, to elucidate the molecular mechanisms regulating tau phosphorylation *in vivo*, we determined the state-dependent activity profile of glycogen synthase kinase 3 beta (GSK3-beta), cyclin dependent kinase 5 (cdk5), stress-activated protein kinase/Jun-amino-terminal kinase (SAPK/JNK) and mitogen activated protein kinase/extracellular regulated protein kinase (MAPK/ERK) during hibernation.

## Results

### Characterisation of site specific, reversible tau phosphorylation

The hibernation-related tau phosphorylation was analysed immunohistochemically and by Western blot using different phosphosite-specific tau antibodies. A summary of the investigated tau phosphorylation sites and the applied antibodies is shown in Figure 1.

**Figure 2:**
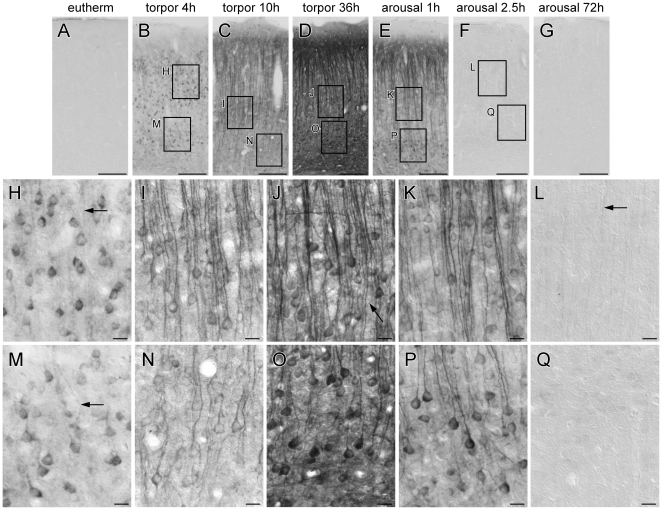
Immunohistological analysis of tau phosphorylation in the neocortex of Syrian hamsters (*Mesocricetus auratus*). The monoclonal antibody AT8 was used to determine the extent of tau phosphorylation in different states of hibernation. A–G: low magnification images showing the reversible phosphorylation pattern. Note that after the entrance into torpor the labelling in the apical dendrites and the band of Bechterew appears a few hours later than in the cell bodies of the pyramidal cells. H–L and M–Q: high magnification images corresponding to the insets in the images B–F and show the staining of pyramidal cells in detail. The labelling of apical dendrites is present very early after the entrance into torpor (arrow in H and M) and disappears at last during arousal (arrow in L). A few basal dendrites were labelled in late torpor (arrow in J). Scale bars: A–G, 200 µm; H–Q, 20 µm.

Generally, tau protein is highly phosphorylated during torpor states and phosphorylation levels decrease after arousal as demonstrated in Figure 2 where the monoclonal antibody AT8 recognizing tau protein phosphorylated at S202/T205 was applied to analyse generation and distribution of phospho-tau during hibernation in the neocortex of Syrian hamsters. Euthermic animals are characterised by a complete absence of AT8 immunoreactivity (Figure 2A) whereas already four hours after entry into torpor a marked increase of tau phosphorylation (Figure 2B) was observed. During progression of torpor state immunolabelling showed a constant increase (Figure 2B–D). Phosphorylated tau protein was first observed in the cell body and the adjacent part of the apical dendrite. However, after a more prolonged time in the state of torpor labelling extended further away from the cell body into the band of Bechterew. In late torpor, a particularly intense labelling was observed in the apical dendrites and cell bodies of many pyramidal cells in layer II and IV (Figure 2D). Noteworthy, the fibres in the band of Bechterew at the upper lamina of layer II were also strongly labelled. A rapid decline in AT8-immunoreactivity was determined after induced arousal. For instance, as early as 1 hour after induction of arousal no phospho-tau was detectable in the cell bodies in layer II and in the band of Bechterew (Figure 2E). Furthermore, there was no labelling except for some apical dendrites as short as 2.5 hours after induction of arousal (Figure 2F). A complete reversal of AT8 immunoreactivity was observed 72 hours after induced arousal (Figure 2G).

**Figure 3:**
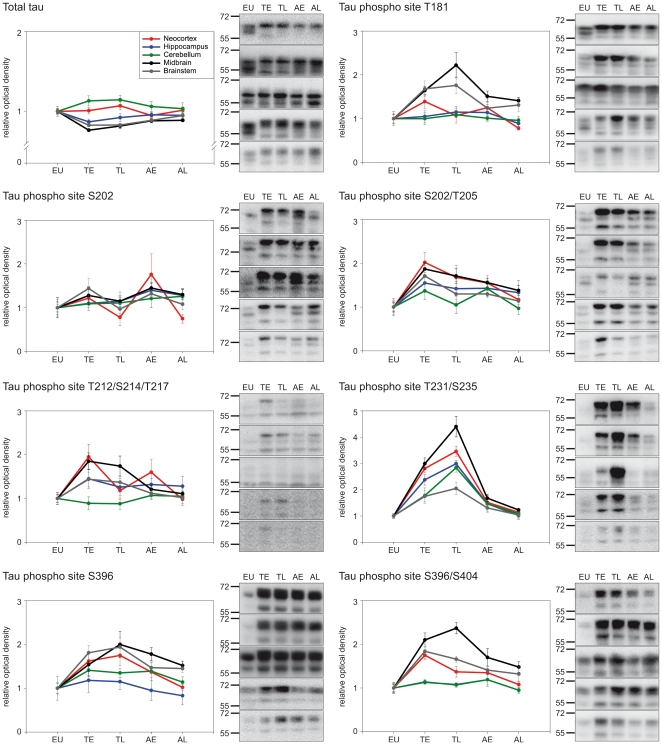
Brain region specific, hibernation-state dependent tau phosphorylation in arctic ground squirrels (*Spermophilus parryii*). Using various phospho site-specific antibodies we analysed the degree of tau phosphorylation by Western blot in brain extracts of neocortex, hippocampus, cerebellum, brainstem and the midbrain including the hypothalamus. The diagrams show the alteration in tau phosphorylation (mean ± SE) in animals of five different hibernation-states; euthermic animals (EU; n = 9), animals in early torpor (TE; n = 5), animals in late torpor (TL; n = 5), animals sampled shortly after spontaneous arousal (AE; n = 7) and animals sampled later after spontaneous arousal (AL; n = 5). The corresponding Western blots are displayed on the right showing representatively the immunoreactivity of the applied antibodies in the investigated brain regions (from top to bottom: neocortex, hippocampus, cerebellum, brainstem, midbrain. The approximate molecular weights (in kDa) are indicated on the left of the panel.

**Figure 4:**
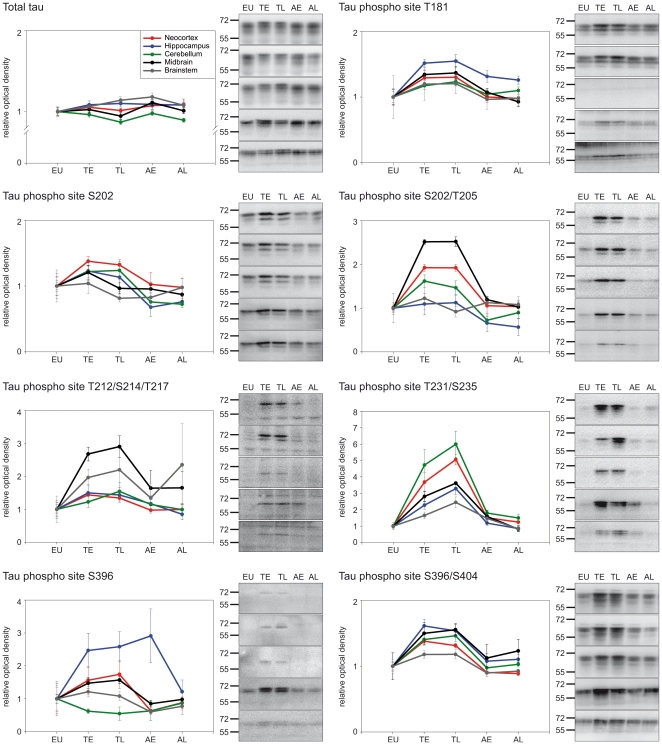
Brain region specific, hibernation-state dependent tau phosphorylation in Syrian hamsters (*Mesocricetus auratus*). Using various phospho site-specific antibodies we analysed the degree of tau phosphorylation by Western blot in brain extracts of neocortex, hippocampus, cerebellum, brainstem and the midbrain including the hypothalamus. The diagrams show the alteration in tau phosphorylation (mean ± SE) in animals of five different hibernation-states; euthermic animals (EU; n = 3), animals in early torpor (TE; n = 5), animals in late torpor (TL; n = 5), animals sampled shortly after induced arousal (AE; n = 3) and animals sampled later after induced arousal (AL; n = 4). The corresponding Western blots are displayed on the right showing representatively the immunoreactivity of the applied antibodies in the investigated brain regions (from top to bottom: neocortex, hippocampus, cerebellum, brainstem, midbrain. The approximate molecular weights (in kDa) are indicated on the left of the panel.

**Figure 5:**
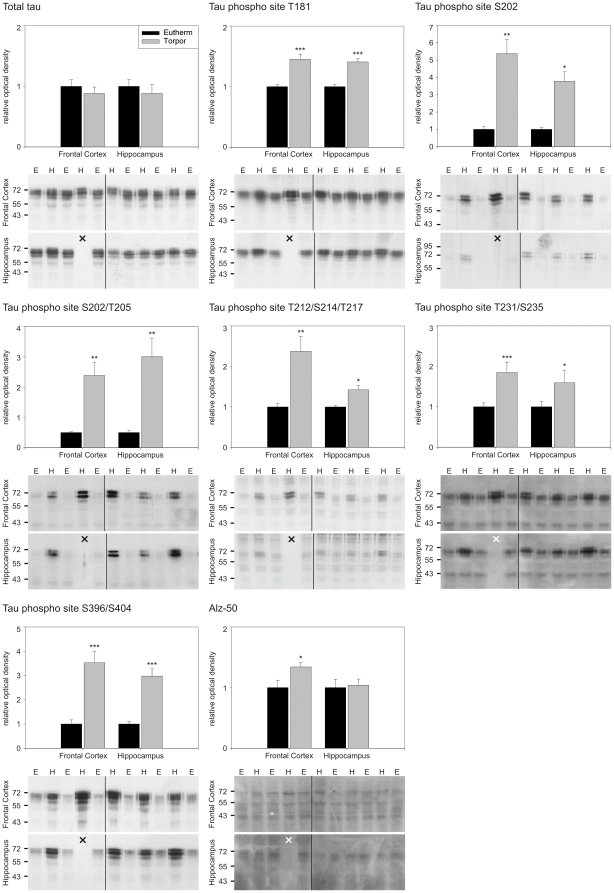
Characterisation of hibernation-state dependent tau phosphorylation in American black bears (*Ursus americanus*). We analysed tau status in the frontal cortex and hippocampus of summer active and hibernating black bears by Western blot using phospho-site-specific and conformation-specific antibodies. The histograms show the relative change in tau phosphorylation of hibernating animals in the frontal cortex (euthermic animals n = 6, hibernating animals n = 5) and hippocampus (euthermic animals n = 6, hibernating animals n = 4). The corresponding Western blots are displayed below, showing the immunoreactivity in frontal cortex (upper panel) and hippocampus (lower panel) (E – euthermic animal; H – hibernating animal). X indicates empty lanes caused by an unavailable hippocampal sample. The approximate molecular weights (in kDa) are indicated on the left. The application of the conformation dependent antibody Alz-50 revealed a significant alteration in tau conformation in the frontal cortex of hibernating animals. Significant alterations are indicated as follows: * p≤0.05; ** p≤0.01; *** p≤0.001.

Antibodies that detect specific phosphorylation sites on tau protein were used to characterise the regional tau phosphorylation pattern during the hibernation cycle by Western blot. In total, seven phosphosites (see Figure 1) were analysed in five (arctic ground squirrel and Syrian hamster) or two (black bear) different brain regions. Brain samples of five different hibernation-states (euthermy, early torpor, long torpor, early arousal and long arousal) were compared in arctic ground squirrels and Syrian hamsters. In black bears, euthermic animals were compared to hibernating animals. The data are summarised in the Figures 3, 4 and 5 and Tables 1 and 2. A number of differences regarding the degree and the kinetics of tau phosphorylation between species, phosphosites, brain regions and hibernation-states were observed.

**Table 1:** Synopsis of hibernation dependent tau phosphorylation in arctic ground squirrels (*Spermophilus parryii*) and Syrian hamsters (*Mesocricetus auratus*).

*Spermophilus parryii*							*Mesocricetus auratus*						
Phosphorsite	Brain region	increase factor				p value(ANOVA)	Phosphorsite	Brain region	increase factor				p value(ANOVA)
		TE	TL	AE	AL				TE	TL	AE	AL	
total tau	Neocortex	1,01	1,07	0,95	1,01	0,4124	total tau	Neocortex	1,05	1,01	1,07	1,09	0,4960
	Hippocampus	0,87	0,92	0,96	0,95	0,1592		Hippocampus	1,08	1,10	1,09	1,08	0,8020
	Cerebellum	1,13	1,15	1,06	1,03	0,4731		Cerebellum	0,96	0,87	0,98	0,89	*0,0110*
	Brainstem	0,83	0,83	0,89	0,95	*0,0001*		Brainstem	1,06	1,14	1,18	1,08	0,0542
	Midbrain	0,76	0,82	0,88	0,89	*0,0001*		Midbrain	1,03	0,95	1,11	1,01	0,0753
T181	Neocortex	1,39	1,07	1,25	0,78	0,1005	T181	Neocortex	1,29	1,30	1,00	0,98	*0,0007*
	Hippocampus	1,05	1,15	1,14	0,89	0,8159		Hippocampus	1,52	1,55	1,31	1,26	*0,0041*
	Cerebellum	1,00	1,09	1,01	0,96	0,9440		Cerebellum	1,17	1,23	1,04	1,10	0,0710
	Brainstem	1,68	1,76	1,24	1,31	*0,0054*		Brainstem	1,20	1,20	0,96	0,98	0,8378
	Midbrain	1,64	2,22	1,51	1,41	*0,0001*		Midbrain	1,34	1,37	1,07	0,93	*0,0062*
S202	Neocortex	1,22	0,78	1,76	0,75	0,1002	S202	Neocortex	1,38	1,32	1,03	0,98	0,0740
	Hippocampus	1,10	1,15	1,40	1,28	0,2664		Hippocampus	1,23	1,13	0,68	0,76	0,1205
	Cerebellum	1,09	1,11	1,21	1,26	0,8072		Cerebellum	1,22	1,24	0,76	0,72	*0,0044*
	Brainstem	1,44	0,97	1,32	1,08	0,1001		Brainstem	1,04	0,81	0,83	0,98	0,7086
	Midbrain	1,28	1,15	1,45	1,30	0,4275		Midbrain	1,21	0,96	0,96	0,87	0,1769
S202/	Neocortex	2,02	1,68	1,55	1,17	*0,0089*	S202/	Neocortex	1,93	1,92	1,06	1,04	*0,0001*
T205	Hippocampus	1,55	1,42	1,43	1,33	0,1796	T205	Hippocampus	1,10	1,13	0,66	0,57	0,2200
	Cerebellum	1,37	1,05	1,42	0,97	0,3137		Cerebellum	1,62	1,47	0,72	0,90	*0,0019*
	Brainstem	1,71	1,30	1,30	1,14	*0,0106*		Brainstem	1,22	0,92	1,13	1,08	0,8340
	Midbrain	1,87	1,71	1,56	1,38	*0,0002*		Midbrain	2,52	2,52	1,20	1,03	*0,0001*
T212/	Neocortex	1,95	1,18	1,60	0,98	*0,0224*	T212/	Neocortex	1,44	1,34	0,97	1,00	*0,0007*
S214/	Hippocampus	1,45	1,26	1,32	1,28	0,3782	S214/	Hippocampus	1,50	1,44	1,17	0,85	0,1852
T217	Cerebellum	0,89	0,88	1,07	1,05	0,7015	T217	Cerebellum	1,23	1,55	1,15	0,99	0,4283
	Brainstem	1,44	1,37	1,12	1,02	0,1299		Brainstem	1,97	2,20	1,34	2,35	0,6893
	Midbrain	1,85	1,74	1,21	1,11	*0,0004*		Midbrain	2,68	2,91	1,64	1,65	*0,0114*
T231/	Neocortex	2,81	3,46	1,52	1,14	*0,0001*	T231/	Neocortex	3,68	5,06	1,51	1,25	*0,0001*
S235	Hippocampus	2,38	2,99	1,47	1,02	*0,0001*	S235	Hippocampus	2,28	3,29	1,19	0,85	*0,0001*
	Cerebellum	1,78	2,84	1,46	1,09	*0,0001*		Cerebellum	4,72	5,99	1,80	1,50	*0,0004*
	Brainstem	1,75	2,05	1,31	1,04	*0,0002*		Brainstem	1,65	2,45	1,47	0,84	*0,0019*
	Midbrain	2,99	4,40	1,68	1,23	*0,0001*		Midbrain	2,81	3,62	1,59	0,82	*0,0001*
S396	Neocortex	1,62	1,75	1,37	1,02	*0,0051*	S396	Neocortex	1,56	1,74	0,63	0,88	0,2731
	Hippocampus	1,18	1,15	0,95	0,83	0,8978		Hippocampus	2,47	2,58	2,91	1,21	0,0978
	Cerebellum	1,41	1,35	1,39	1,14	0,2388		Cerebellum	0,62	0,55	0,63	0,87	0,0709
	Brainstem	1,81	1,94	1,47	1,45	*0,0002*		Brainstem	1,21	1,08	0,60	0,77	0,1551
	Midbrain	1,54	2,00	1,78	1,52	*0,0037*		Midbrain	1,47	1,57	0,85	0,98	*0,0025*
S396/	Neocortex	1,75	1,37	1,35	1,08	*0,0007*	S396/	Neocortex	1,38	1,31	0,90	0,89	*0,0001*
S404	Hippocampus	1,13	1,08	1,19	0,95	0,4571	S404	Hippocampus	1,61	1,54	1,08	1,11	*0,0006*
	Cerebellum	1,14	1,07	1,19	0,95	0,4744		Cerebellum	1,40	1,46	0,97	1,03	*0,0002*
	Brainstem	1,84	1,66	1,41	1,32	*0,0013*		Brainstem	1,18	1,18	0,90	0,92	*0,0115*
	Midbrain	2,10	2,37	1,70	1,48	*0,0001*		Midbrain	1,50	1,55	1,12	1,23	0,1389

The table shows the increase of tau phosphorylation at specific sites in different brain regions during the hibernation cycle (TE – early torpor; TL – late torpor; AE – early arousal; AL – late arousal). Data are based on Western blot experiments. Increase factors are related to the level of phosphorylation in euthermic animals. Italic p-values indicate significant alterations (p≤0.05).

**Table 2:** Brain region specific comparison of hibernation related tau phosphorylation.

*Spermophilus parryii*			*Mesocricetus auratus*			*Ursus americanus*		
Brain region	altered phospho site	p value(ANOVA)	Brain region	altered phospho site	p value(ANOVA)	Brain region	altered phospho site	p value
Neocortex	S202/T205	0.0089	Neocortex	T181	0.0007	Neocortex	T181	0.0001 ¶
	T212/S214/T217	0.0224		S202/T205	0.0001		S202	0.0013 †
	T231/S235	0.0001		T212/S214/T217	0.0007		S202/T205	0.0043 ¶
	S396	0.0051		T231/S235	0.0001		T212/S214/T217	0.0011 †
	S396/S404	0.0007		S396/S404	0.0001		T231/S235	0.0004 ¶
Hippocampus	T231/S235	0.0001	Hippocampus	T181	0.0041		S396/S404	0.0004 †
Cerebellum	T231/S235	0.0001		T231/S235	0.0001	Hippocampus	T181	0.0003 †
Brainstem	T181	0.0054		S396/S404	0.0006		S202	0.0154 †
	S202/T205	0.0106	Cerebellum	S202	0.0044		S202/T205	0.0095 ¶
	T231/S235	0.0002		S202/T205	0.0019		T212/S214/T217	0.0190 †
	S396	0.0002		T231/S235	0.0004		T231/S235	0.0381 †
	S396/S404	0.0013		S396/S404	0.0002		S396/S404	0.0001 †
Midbrain	T181	0.0001	Brainstem	T231/S235	0.0019			
	S202/T205	0.0002		S396/S404	0.0151			
	T212/S214/T217	0.0004	Midbrain	T181	0.0062			
	T231/S235	0.0001		S202/T205	0.0001			
	S396	0.0037		T212/S214/T217	0.0114			
	S396/S404	0.0001		T231/S235	0.0001			
				S396	0.0025			

The table summarizes the significantly altered (p≤0.05) phospho sites of tau protein during hibernation in all analysed species.

¶ - Mann-Whitney rank sum Test.

† - t-Test.

The analysis of results from arctic ground squirrels (Figure 3) and Syrian hamsters (Figure 4) revealed the most detailed findings since in contrast to black bears additional hibernation-states and brain regions were studied resulting in information about the kinetics of tau phosphorylation/dephosphorylation during torpor and arousal periods. A comprehensive summary of the results is listed in Table S1. All of the analysed tau phosphosites are affected during hibernation and showed increased degree of phosphorylation in torpor states with exception of the S202 site in arctic ground squirrels, where no alterations were found. Overall, in the late arousal samples, tau phospho levels had decreased and showed degrees comparable to those of euthermic, non-hibernating animals with slight variations regarding the analysed brain regions (Table 2). The highest degree of tau phosphorylation was observed in the neocortex and the midbrain, including the hypothalamus. In both regions most of the analysed phosphosites showed hibernation-state dependent reversible tau phosphorylation, whereas the analysis of hippocampus, brainstem and cerebellum revealed a more complex pattern.

The tau phosphosite T231/S235 was the only site showing reversible phosphorylation in all brain regions analysed. Interestingly, for this site we also observed aberrant phosphorylation kinetics. In general, tau phosphorylation did not further increase in late torpor. However, phospho levels at T231/S235 were significantly elevated in late torpor compared to early torpor. This increase was detected in both arctic ground squirrels and Syrian hamsters indicating a conserved characteristic of this particular site.

In black bears sampled during hibernation all seven of the investigated phosphosites showed an increased phosphorylation level compared to summer active animals, both in frontal cortex and hippocampus (Figure 5, Table 2).

We applied the Alz-50 antibody that specifically detects a particular tau conformation, typically for PHF-tau [35]. The analysis of protein extracts from both arctic ground squirrels and Syrian hamsters revealed no conformational change (data not shown). In contrast, in the frontal cortex of hibernating black bears we found a significant increase of immunoreactivity (Figure 5). This finding shows that in black bears an elevated tau phosphorylation is associated with conformational changes, which are typically related to pathological alterations in neurons.

### Analysis of tau expression

Tau protein expression was analysed by Western blot using a phospho-independent tau antibody. The results are listed in Table 1 and shown in Figures 3, 4 and 5. There was no overall pattern of altered tau protein expression. Nevertheless, tau protein expression was decreased in the brainstem and midbrain of torpid arctic ground squirrels compared to euthermic animals. It was also reduced in the cerebellum of torpid Syrian hamsters. Tau expression reverted to euthermic levels after arousal except for the midbrain of arctic ground squirrels where expression was still reduced in late arousal. There were no changes of tau protein expression in black bears.

**Figure 6:**
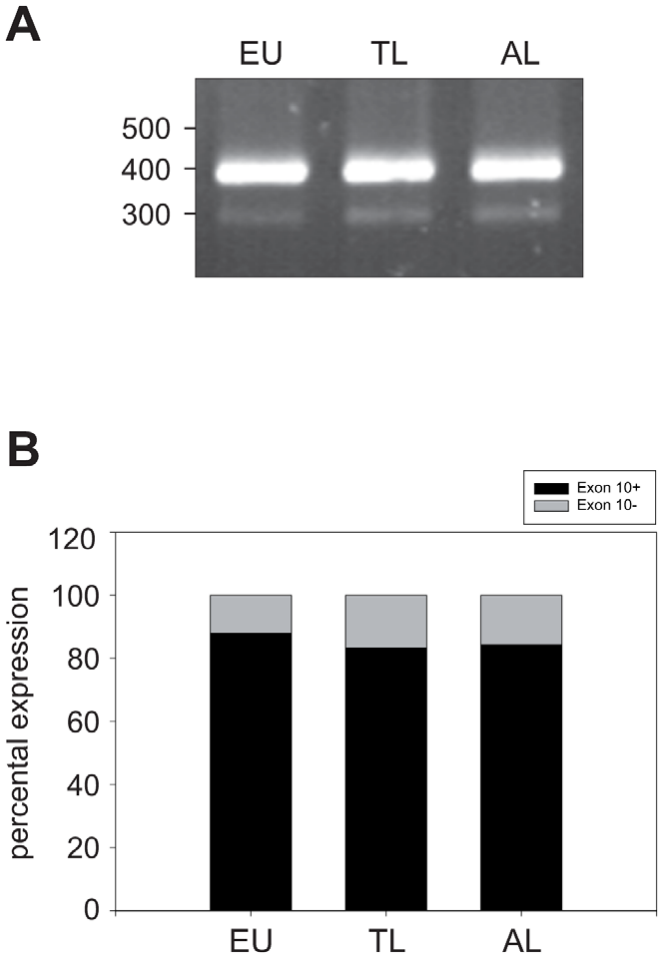
Analysis of hibernation-state dependent alternative splicing of tau exon 10 in arctic ground squirrels (*Spermophilus parryii*) (euthermic n = 4; torpor long n = 4, arousal long n = 4). Species-specific primers were used to determine the expression of tau exon 10 in different states of hibernation. A: Two distinct PCR products with a size of about 390 bp and 290 bp were obtained corresponding to the predicted PCR product size depending on the presence or absence of exon 10 (representative set). B: The ratio of expressed tau isoforms including and lacking exon 10, respectively, was determined by analysing the optical density of both PCR products. The expression was calculated as percentage related to the summative intensity of all bands (set to 100 %). The tau isoform expression pattern is significantly altered during hibernation (p = 0.0043; ANOVA) and the expression of tau isoforms including exon 10 is significantly decreased in the state of torpor (p = 0.0081; t-Test) and after arousal (p = 0.002; t-Test).

Regarding tau mRNA isoform expression we focused on the analysis of a potentially altered splicing of exon 10 that encodes for an additional microtubule binding repeat and thereby alters the binding capacity of tau. The analysis was performed in arctic ground squirrels (EU, n = 4; TL, n = 4, AL, n = 4) whereas prior to the isoform expression analysis Tau gene transcripts of were cloned and sequenced (GenBank accession number: FJ609677). Based on the determined sequence, species-specific primers were designed binding in exon 7 and exon 11 respectively. The PCR revealed two distinct PCR products with a size of about 390 bp and 290 bp corresponding to the predicted PCR product size depending on the presence or absence of exon 10 thereby demonstrating the expression of both 3-repeat and 4-repeat tau isoforms in adult arctic ground squirrels (Figure 6A). To determine the hibernation state related ratio of expressed tau isoforms including and lacking exon 10 respectively the optical density of the PCR products was analysed (Figure 6B). The results show a significantly altered tau isoform expression pattern during hibernation (p = 0.0043; ANOVA). And the expression of tau isoforms including exon 10 is significantly decreased in the state of torpor (p = 0.0081; t-Test) and after arousal (p = 0.002; t-Test).

### Phospho-protein stain

**Figure 7:**
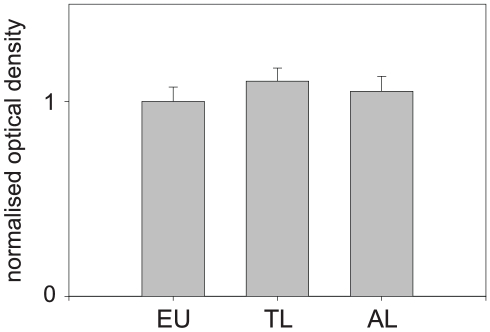
Hibernation-state dependent formation of phospho-proteins in arctic ground squirrels (*Spermophilus parryii*) (euthermic n = 4; torpor long n = 4, arousal long n = 4). We found no evidence for a state-dependent alteration of phospho-protein content (p = 0.618; ANOVA).

The formation of phospho-proteins was determined in neocortical brain homogenates of arctic ground squirrels (EU, n = 4; TL, n = 4, AL, n = 4). There were no differences regarding the levels of phospho-proteins during hibernation (Figure 7).

### Analysis of tau phosphorylation mechanisms

#### Tau phosphate net turnover assays

To determine whether tau phosphorylation during hibernation is specifically regulated or a passive process driven by temperature-dependent alterations of kinase- and phosphatase activities, we analysed the net turnover of phosphate in tau protein at different incubation temperatures. This assay is designed to determine both phosphorylation and dephosphorylation of tau by assessing the incorporation of radioactively labelled phosphate residues into the tau protein. Therefore, the assay reflects the result of kinase and phosphatase activities in the analysed brain extracts. The result of enzyme activities was assessed in cortical brain samples of arctic ground squirrels (EU, n = 4; TL, n = 4, AL, n = 4).

**Figure 8:**
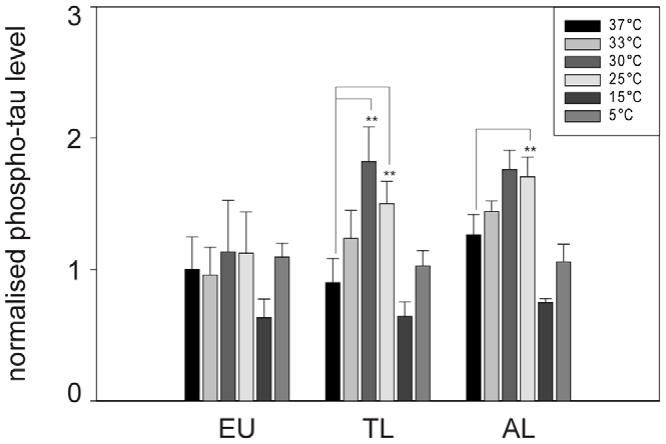
Temperature dependent phosphate net turnover of tau protein in different hibernation-states. The summative effect of kinase and phosphatase activities was assessed in cortical brain samples of arctic ground squirrels (*Spermophilus parryii*) (euthermic n = 4; torpor long n = 4, arousal long n = 4). Samples were incubated at various temperatures and times (37°C, 5 min; 33°C, 7 min; 30°C, 8.5 min; 25°C, 11 min; 15°C, 16 min; 5°C, 21 min; 0°C, 23.5 min). ANOVA was used to perform the statistical analysis and significant alterations are highlighted (** p≤0.01).

First, we analysed the phosphate net turnover at various temperatures (Figure 8). Reaction times for each temperature were previously optimised using samples of euthermic animals (37°C, 5 min; 33°C, 7 min; 30°C, 8.5 min; 25°C, 11 min; 15°C, 16 min; 5°C, 21 min; 0°C, 23.5 min). No signal was detected at 0°C, and, therefore, this reading point was excluded from further experiments.

Tau protein phosphate net turnover was significantly altered (p≤0.01, ANOVA) in both torpid and aroused animals as compared to euthermic animals. Most interestingly, there was no general shift towards increased phosphorylation in torpor and arousal. We rather determined a temperature dependent variation in tau phosphorylation in these hibernation states. The most intense phosphate intake was observed at 30°C in torpid animals resulting in an almost doubled phospho-tau degree.

**Figure 9:**
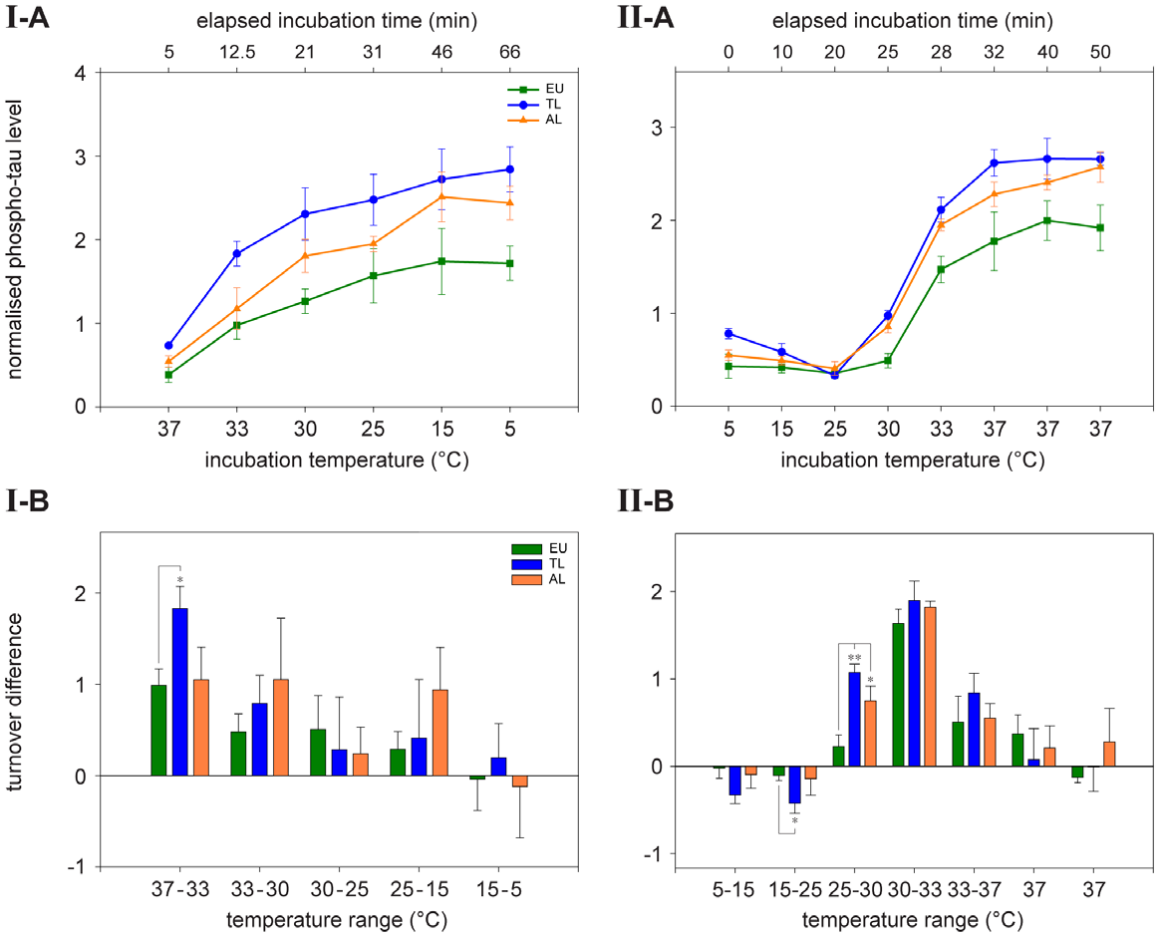
Hibernation-state related tau phosphate net turnover in a decreasing (I-A, I-B) and increasing (II-A, II-B) temperature gradient. The effect of enzyme activities was analysed in cortical brain samples of arctic ground squirrels (*Spermophilus parryii*) in different hibernation-states (euthermic n = 4; torpor long n = 4, arousal long n = 4). I-A and I-B: reactions were started at 37°C and aliquots were taken successively from the samples after incubation at different temperatures. II-A and II-B: Samples were pre-incubated 15 minutes at 33°C and instantly chilled on ice. Record of reaction was started at 5°C and aliquots were taken successively from the samples after incubation at different temperatures. Progress of reactions is shown in figures I-A and II-A while the differences in tau phosphate net turnover between two reading points (Δ) is shown in diagram I-B and II-B. Data indicating hibernation-state dependent differences in enzyme kinetics, consequently resulting in altered degree of tau phosphorylation after termination of reaction. Furthermore, these results demonstrate that enzyme kinetics are differentially regulated dependent on hibernation-state and temperature. Statistical analyses were performed with Student's t-Test and significant alterations are highlighted (** p≤0.01, * p≤0.05).

To further analyse this temperature dependency, we measured rate of tau phosphate net turnover at a series of temperatures that simulated the entry into torpor and arousing *in vitro*. Brain samples were taken from euthermic, torpid and aroused animals and subjected to a decreasing and an increasing temperature gradient. As shown in Figure 9, temperature dependency of phosphate incorporation showed characteristic and significant differences between tissues taken from animals at different stages during the hibernation cycle. Tissue taken from torpid animals was most sensitive to the temperature shift, followed by tissue from aroused and euthermic animals. In torpid animals, for example, the phospho-tau level was already higher after an incubation time of 12.5 min compared to that of euthermic animals after the total incubation time of 66 min. Differences between the stages of the hibernation cycle were most pronounced at the transition from 37 to 33°C for the cooling down phase (Figure 9 I-B) and 25 to 33°C for the warming up phase (Figure 9 II-B). During re-warming, phosphate incorporation showed a biphasic response. There was an initial dephosphorylation between 5°C and 25°C (p≤0.05, t-Test), followed by enhanced phosphorylation at temperatures above 25°C (p≤0.05, t-Test), (Figure 9 II-A). The prolonged incubation at 37°C at the end of the simulated arousal did not yield in decreased, but rather in constant phospho-tau levels indicating superordinate regulatory mechanisms for the rapid dephosphorylation of tau during the arousal of animals.

### Profile of activity state of tau kinases in hibernating animals

We determined brain tissue activity of the major tau kinases glycogen synthase kinase 3 beta (GSK3-beta) cyclin dependent kinase 5 (cdk5), stress-activated protein kinase/Jun-amino-terminal kinase (SAPK/JNK), and mitogen activated protein kinases/extracellular regulated protein kinase (MAPK/ERK). Activity of these kinases is regulated by phosphorylation, which increases enzymatic rates, except for GSK3-beta, where phosphorylation of residue S9 is inhibitory [36].

**Figure 10:**
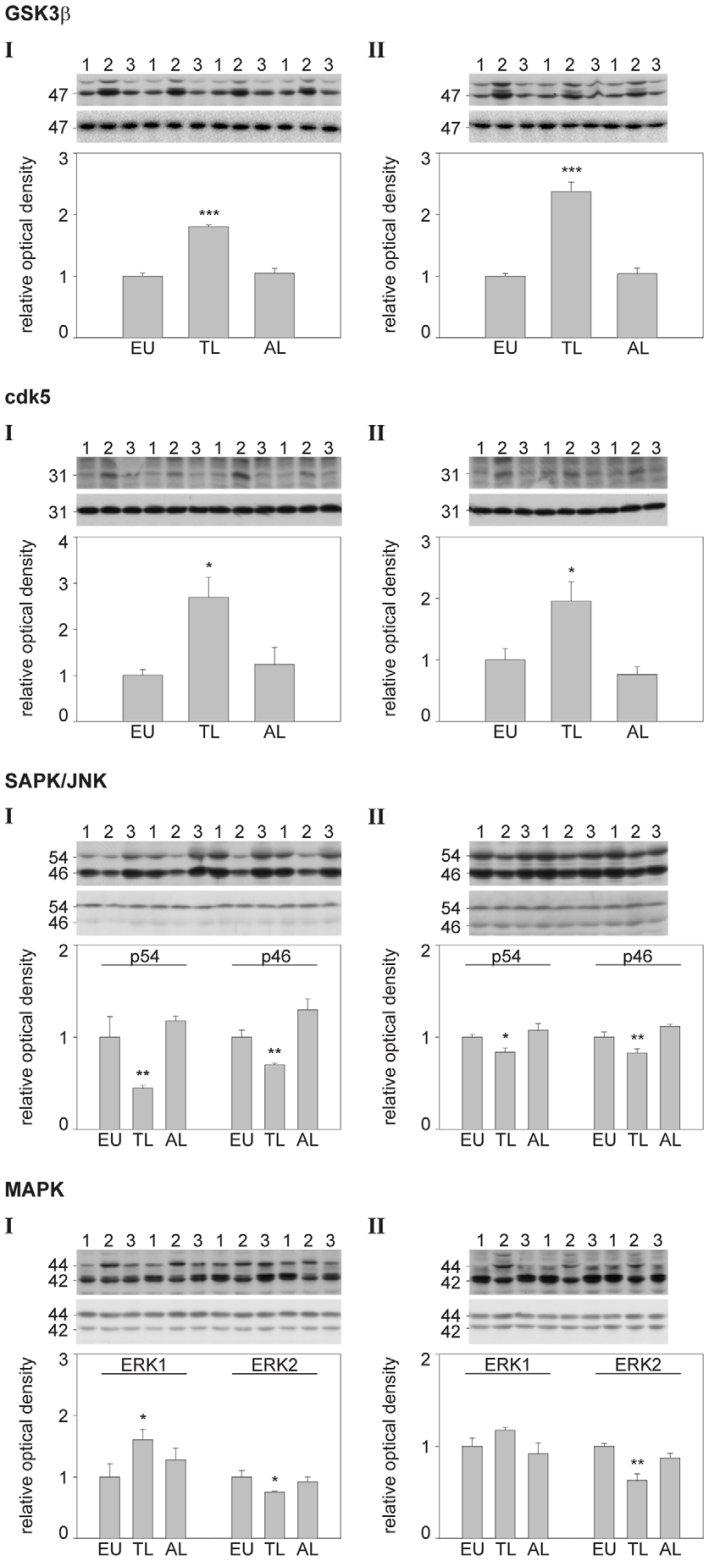
Activity state profile of tau kinases in hibernating animals. Using phospho-specific antibodies we analysed the activation status in neocortical brain extracts of *Spermophilus parryii* (I) and *Mesocricetus auratus* (II) respectively. The diagrams show the alteration in kinase phosphorylation (mean ± SE) in euthermic animals (EU), animals in late torpor (TL), and animals sampled after arousal (AL). The lanes of the corresponding Western blot panels are labelled with 1 (euthermic animals), 2 (torpid animals) and 3 (aroused animals) and show the detection of the phosphorylated enzyme (upper panel) as well as its expression level (lower panel) using phospho-independent antibodies. The approximate molecular weights (in kDa) are indicated on the left of the panel. Statistical analyses were performed using ANOVA and significant alterations are indicated as follows: * p≤0.05; ** p≤0.01; *** p≤0.001.

Phosphorylation of GSK3-beta and cdk5 was significantly increased in torpid animals, indicating inhibition of GSK3-beta and activation of cdk5 activity during torpor (Figure 10). Phosphorylation of SAPK/JNK was significantly decreased with respect to p54 and p46, suggesting a reduction in SAPK/JNK activity during torpor. The analysis of the MAPK/ERK activity state revealed divergent results. In torpid animals ERK1 (p44) is highly phosphorylated, whereas ERK2 (p42) shows a decreased phosphorylation. All alterations in kinase activity status were completely reversed after arousal.

## Discussion

### PHF-like tau-phosphorylation is a general characteristic of mammalian hibernation

In ground squirrels, the microtubule-associated brain protein tau enters a hyperphosphorylated state during hibernation that is fully reversed when animals periodically arouse to normal levels of metabolism and brain temperature [24], [29]. Since these changes occur spontaneously and seemingly without pathology, mammalian hibernation may be a useful model for investigating physiological aspects of tau protein phosphorylation *in vivo*.

Hibernation is a behavioural and physiological adaptation in mammals in which the need for energy is profoundly decreased during conditions of current or anticipated famine. Hibernating animals enter a state of reduced metabolism and decreased body temperature called torpor that can continue for several days to several weeks in small mammals and several months in large mammals. Hibernation is found in a variety of species in several different orders [37]. This behaviour is not restricted to specific geographical areas and it is not a feature of a particular evolutionary stage of development. It rather can be observed in rodents inhabiting the Arctic, where core body temperature can decrease to below the freezing point [38] and in primates living in tropical regions [39]. In small mammals torpor is interrupted regularly by arousal episodes, which are brief (<20 hours) returns to normal levels of metabolic rate and body temperature. The biological relevance of these arousals is still not understood. However, they are likely important for restoration of some physiological or neurological capacity or even be necessary to prevent damage [24], [40].

Mammalian hibernation is thought to be based on similar physiological mechanisms [41], [42] and a consequence of alternative regulation of general physiological programmes by the differential expression of existing genes. Hence, regulated hypometabolism may be supposed as a basal physiological function of mammals.

Here we analysed the hibernation-state dependent tau phosphorylation in arctic ground squirrels (*Spermophilus parryii*), Syrian hamsters (*Mesocricetus auratus*) and black bears (*Ursus americanus*). These species were selected to determine whether tau phosphorylation is a general, hibernation-related phenomenon and whether species that hibernate with different body temperatures and patterns show the same patterns of phosphorylation change.

Arctic ground squirrels and black bears are obligate hibernators. Hibernation of these species is controlled by an endogenous circannual rhythm. Although referring to the same hibernation category, the physiological parameters of these species in torpor differ substantially.

Under natural conditions the hibernation season of arctic ground squirrels starts in September and ends in April. The basic metabolic rate of a torpid animal is decreased to only 2% compared to euthermic conditions [43].

With an extent of four to seven months the duration of the hibernation season of black bears is similar to that of arctic ground squirrels. However, hibernation of black bears is continuous, i.e. in contrast to arctic ground squirrels it is not interrupted by spontaneous arousals. They rather abide the entire hibernation season in a den without drinking, eating, urinating and defecating [44], [45]. All animal rates of metabolism are only slightly narrowed. The minimum metabolic rate of a hibernating bear is lowered to about 25% compared to non-hibernating resting state and body temperature usually declines to no lower than 30°C [46].

Syrian hamsters are referred to as permissive hibernators, i.e. hibernation is an optional response to temporary non-optimal environmental conditions. Very little is known about the behaviour and physiology of wild Syrian hamsters. However, limited food supply, low ambient temperature and a reduced photoperiod may trigger animals to enter hibernation. Hence, these parameters are critical elements for the induction of hibernation in Syrian hamsters under laboratory conditions [47]. Once entered into hibernation animals display a hibernation pattern similar to that of arctic ground squirrels. Torpor bouts alternate with spontaneous arousals were animals revert to euthermic state. Values of basic metabolic rate, body temperature and torpor bout duration differ depending on the experimental conditions. The body temperature decreases to a range of 1–2°C above ambient temperature that in the most applied setups varies between 4°C and 8°C and basic metabolic rate may be reduced to about 2.5% when compared to euthermy [48].

The interspecies comparison of hibernation-related tau phosphorylation was one major element of this study. Up to now hibernation related PHF-like tau phosphorylation was reported for European ground squirrels [24] and recently for arctic ground squirrels [28], [29]. An increased phosphorylation of tau at the site S202/T205 in association with hibernation was also shown in Syrian hamsters [27].

In the present work we comprehensively describe the formation of PHF–like tau phosphorylation during hibernation in arctic ground squirrels, Syrian hamsters and black bears and thereby strongly substantiate the indication that reversible tau phosphorylation is a fundamental characteristic of the hypometabolic state during mammalian hibernation regardless of the different physiological characteristics of hibernation.

### Regulation of tau-phosphorylation during hibernation involves both hibernation-state specific and passive temperature-driven mechanisms

In addition to hibernation, increased tau phosphorylation has also been reported during starvation [49]–[51], anaesthesia [52], [53], and cold water stress [54], conditions which all are associated with a reduced body temperature. Planel and co-workers suggested that due to differences in temperature dependency of kinetics for tau kinases and tau phosphatases, lower body temperature ultimately results in increased levels of phospho-tau [51]. Therefore, in our study, we addressed the question whether the observed increase in tau-phosphorylation during hibernation might simply be due to such passive, temperature-driven mechanism, or if in addition, hibernation-related specific regulatory mechanism might be involved.

The results of the phospho-protein stain revealed only a tendency towards increased phospho-protein concentration in the state of torpor. Considering the number of potential phospho-proteins and the intense phospho-tau level in hibernation this finding does not support a shift towards a generally increased kinase activity at lower body temperatures and indicates specific regulatory mechanisms in addition. This is corroborated by the results of the net phosphate turnover assay of tau protein. Overall, our results correspond to findings of previous studies showing that a decreased body temperature results in the formation of highly phosphorylated tau [29], [51], [53]. Characteristics of this dependency of tau-phosphorylation on temperature, however, markedly differed, depending on whether tissue was taken from torpid, aroused or euthermic animals. At comparable temperatures, phosphate incorporation into tau was much facilitated in tissue from torpid animals compared to euthermic animals. This indicates the existence of a specific component in the regulation of tau phosphorylation at early stages of hibernation when animals enter into torpor.

### Site-specific phosphorylation and regulation of tau-protein kinases during hibernation

The analysis of site-specific differences in the kinetics of tau phosphorylation and dephosphorylation showed that in general tau phosphosites were already highly phosphorylated in early torpor and the degree of phosphorylation did not increase further in the course of torpor. Nevertheless, the phosphosite T231/S235 is subject to aberrant phosphorylation kinetics. The phospho-degree of this site significantly increased with progression of torpor in arctic ground squirrels and Syrian hamsters, respectively, indicating a substantially different regulation. However, based on the significant overlap with respect to the kinases and phosphatases that are involved in the regulation of phosphorylation/dephosphorylation of the investigated individual sites [55] it is intricate to denote the underlying mechanisms.

Our results do not show any indication of a differentially regulated site-specific dephosphorylation of tau after arousal. We found a significantly elevated phosphorylation after late arousal only in the midbrain for the phosphosites T181 and S396/404 and in the brainstem for S396. In each of the other brain regions investigated all phosphosites showed a level not different from the euthermic state. These results are at variance to those of Su and co-workers who reported that the phosphosites T205, S214 and S396 remain phosphorylated after arousal [29] in arctic ground squirrels.

Our results demonstrate a progressive decrease of phospho-levels from late torpor to early arousal and furthermore to late arousal with site-specific differences in dephosphorylation rates. This is supported by findings demonstrating site-specific dephosphorylation kinetics of tau protein [56].

In the present study we observed an equipollent tau phosphorylation pattern involving phosphosites that have been demonstrated being affected in both early and late stages of AD. The involvement of the tau phosphosites T231/S235 and T212/S214/T217 respectively is remarkable since they are suggested to be directly related to pathological processes [57], [58]. Particularly the generation of the epitope T212/S214/T217 underlies a complex regulation and requires a chronological and site-specific phosphorylation by different kinases [59] and has been considered as typical characteristic of AD.

To elucidate the involvement of individual kinases in the differentially regulated tau phosphorylation, we studied the activity state profile of the tau kinases glycogen synthase kinase 3 beta (GSK3-beta) cyclin dependent kinase 5 (cdk5), stress-activated protein kinase/Jun-amino-terminal kinase (SAPK/JNK) and mitogen activated protein kinases/extracellular regulated protein kinase (MAPK/ERK) in arctic ground squirrels and Syrian hamsters. Interestingly, the results revealed a differentially regulated enzyme activity pattern. Generally, GSK3-beta is supposed to be the primary candidate kinase responsible for tau hyperphosphorylation whereas the other kinases are assumed to modulate tau phosphorylation [60]. In contrast, our results demonstrate an increased phosphorylation of the S9 residue of GSK3-beta indicating an inhibited or at least reduced GSK3-beta activity in torpid animals. In addition, the results are consistent with findings showing inhibition of GSK3-beta in starved mice [51]. GSK3-beta is involved in a variety of physiological processes including the regulation of metabolism. Therefore a differential, hibernation-state dependent GSK3-beta activity is a very likely phenomenon. However, a decreased activity does not correlate with the abnormally high degree of tau phosphorylation.

The phosphorylation of cdk5 at S159, in contrast, indicates an increased activity in the state of torpor [61]. The enzymatic activity of cdk5 is mainly regulated by its binding to a regulatory subunit (p35, p25 or p39). However, since there is no hibernation-state dependent alteration in expression of the regulatory subunit p35 [29] and no formation of p25 [62] we suggest that cdk5 activity underlies a moderate regulation that may directly or indirectly contribute to tau phosphorylation.

The decreased phosphorylation of SAPK/JNK indicates an inhibited activity during torpor. This finding is consistent with results showing decreased SAPK/JNK activity in torpid arctic ground squirrels [63]. On the contrary, in brains of Richardson's ground squirrels (*Spermophilus richardsonii*) no hibernation-related change in JNK activity was found [64].

The MAP-kinases ERK1 (p44) and ERK2 (p42) showed a differentially regulated activity pattern. ERK1 phosphorylation was increased while ERK2 was less phosphorylated in torpid animals. These results are contrary to findings reporting on a decrease of both ERK1 and ERK2 activity during torpor in arctic ground squirrels [63].

To summarise, we found cdk5 and ERK1 positively yet GSK3-beta, SAPK/JNK and ERK2 negatively regulated in torpid animals. The determined kinase activity-state pattern was analogous in both analysed species indicating equivalent regulatory mechanisms. Based on our findings cdk5 and ERK1 may act as kinases that actively phosphorylate tau under physiological conditions. Consequently, since hibernation is not associated with pathological consequences, activation of GSK3-beta and SAPK/JNK as demonstrated in Alzheimer's disease may indicate pathophysiological mechanisms.

### Hypometabolism and increased tau phosphorylation are critical aspects of neurodegenerative disorders

Unravelling the regulation of hypometabolic states such as hibernation are of great significance for the understanding of cellular and molecular aspects of neurodegenerative disorders where hypometabolic states of a “vita minima” precede cell death. As shown by functional brain imaging, these hypometabolic states occur very early during the course of AD, even in presymptomatic stages [65]–[67]; they are a predictor of cognitive decline [68]–[70] and might, thus, be attractive therapeutic targets [71]. A potential role of hypometabolic stages in the pathomechanism of AD is supported by recent data on thyroid disease as a potential risk factor for AD [72]. Hypometabolic states and deficiencies in brain energy metabolism have been proposed as primary events in a pathogenetic chain eventually leading to a hyperphosphorylation of tau [73]–[75] and the whole spectrum of AD pathology [76]–[78].

For that reason, physiological adaptations that are observed at the hypometabolic state in hibernation are potentially analogous to neuronal reactions to a hypometabolism in very early stages of AD.

**Figure 11:**
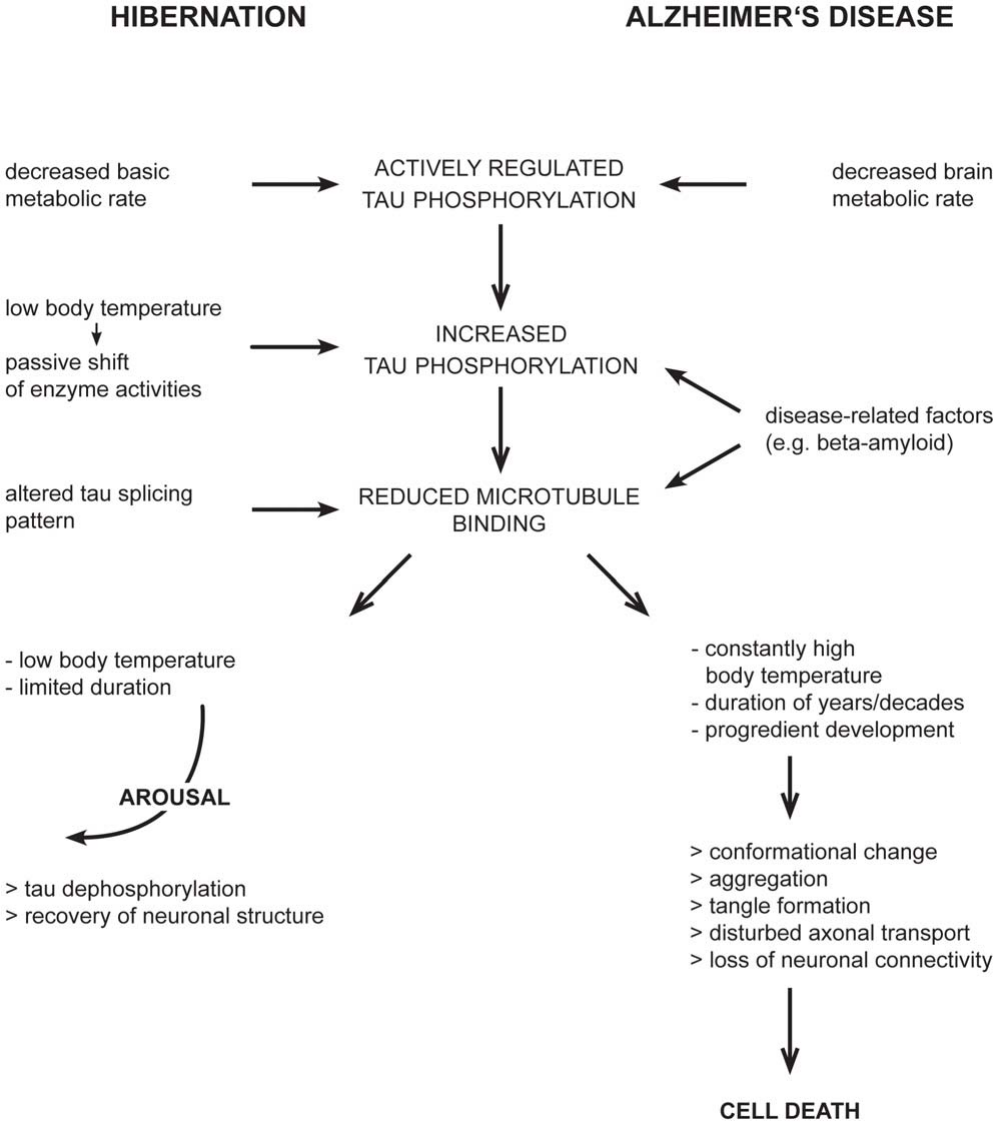
Schematic illustration comparing the cellular consequences of physiological (hibernation) and pathological (AD) tau phosphorylation.

Both, the increased tau phosphorylation and the reduced expression of the four-repeat isoforms result in a decreased microtubule binding capacity of tau protein. This coincidence strongly suggests that this particular condition is one prerequisite for neurons to endure the state of torpor. In this regard the biological relevance of an increased phosphorylation of tau protein in preclinical stages of AD might be reconsidered. It is very likely that particular types of neurons respond to a hypometabolic state with an elevated phosphorylation of tau protein. Hence, this reaction represents a physiological mechanism of the cell and not a primary pathological event [79]. However, in contrast to hibernation, the hypometabolic state is not terminated after a definite time but rather persists and progresses. The phosphorylation of tau protein endures and in the course the actually reversible physiological reaction turns into a pathological event promoted by the large period of time of AD pathogenesis (Figure 11).

In the neocortex of hibernating black bears we found a conformational change of tau protein. This particular interspecies difference might be the result of the considerably elevated body temperature in torpor of bears. *In vitro* studies could demonstrate that the aggregation of tau is inhibited at low temperatures [80]. Since an altered conformation of tau is suggested to impact the propensity of aggregation [15], [16] the change of protein conformation in black bears might reflect a transitional state of a physiological process and thereby highlights the limitation of that particular cellular reaction pattern. A progression may potentially yield in aggregation and tangle formation. This hypothesis is supported by the report of neurofibrillary tangle formation in aged bears [81].

A PHF-like phosphorylation and altered isoform expression of tau protein occurs during torpor in three species of hibernators that differ greatly in body size and in the minimum brain temperatures and metabolic rates they achieve. This phosphorylation is fully reversed when animals return to normal levels of temperature and metabolism whether it is regularly during arousal intervals in small hibernators or seasonally in large hibernators. These findings indicate that a reduced binding capacity of tau may be a precondition to endure the hypometabolic state and reduced tissue temperatures during hibernation. Moreover, the interspecies homogeneity of this reaction pattern suggests that this regulation is subject to a basal physiological mechanism of mammals. Increased neuronal tau phosphorylation in early stages of AD, therefore, may be potentially considered as physiological reaction to a reduced brain metabolic rate. However, as the result of the slow and progressive pathological process in AD, hyperphosphorylated tau protein aggregates to neurofibrillary tangles most likely leading to the degeneration of affected neurons.

PHF-like tau phosphorylation in hibernation is paralleled by an aberrant synaptic plasticity and a loss of memory. Due to the co-occurrence of these elementary attributes of AD hibernating mammals represent a unique and useful model organism to study the relevance and coherencies of particular characteristics of AD pathology. This is of utmost importance for the development of potential strategies for a medical treatment and therapy of this disease.

## Materials and Methods

### Animals and brain tissue preparation

#### Ethics Statement

Only animals in good health were used.

Arctic ground squirrels (*Spermophilus parryii*): Animal protocols were approved by the University of Alaska Fairbanks Institutional Animal Care and Use Committee (IACUC 06-06, 06-25).

Syrian hamsters (*Mesocricetus auratus*): All experimental procedures on animals were carried out in accordance with the European Council Directive of November 24th 1986 (86/609/EEC) and had been approved by the local authorities (T74/05, Regierungspräsidium Leipzig).

American Black bears (*Ursus americanus*): Animal protocols were approved by the University of Alaska Fairbanks Institutional Animal Care and Use Committee (protocols no. 02-39, 02-44, 05-55, 05-57) and USAMRMC Animal Care and Use Review Office (proposal Number 05178001).

### Brain tissue preparation

#### Arctic ground squirrels (*Spermophilus parryii*)

Arctic ground squirrels (n = 31) were trapped during August in the Alaska Range or on the North Slope of Alaska near Toolik Lake and transferred to University of Alaska Fairbanks. The animals were housed individually and provided with Mazuri Rodent Chow sunflower seeds, carrots, and apple slices and provided with water *ad libitum*. In September, temperature-sensitive radiotransmitters (Data Sciences International, St. Paul, MI, USA) were implanted into the peritoneal cavity of each animal (body mass range 650–950 g). Before implant, transmitters were sealed in heat shrink tubing and triple coated in Elvax (Minimitter Inc., Sunriver, OR) creating a package weight of 17–20 g, calibrated to the nearest 0.1°C at 0 and 20°C against a precision mercury glass thermometer, and gas sterilized [82]. Animals were transferred to a chamber held at 2°C with an 8∶16 h light dark cycle where they entered hibernation. Body temperatures of each animal were recorded to computer each 15 minutes through individual radio receivers. After animals spontaneously ended hibernation, their reproductive and molt status was assessed once per week.

In this study, animals were compared at five stages of their annual cycle. Euthermic animals (EU; n = 9) that had ended hibernation and been continuously at high body temperatures for 1–2 months were sampled in May and June after having gone through their reproductive phase and entered spring molt. Animals in hibernation were sampled in mid winter when 3–5 days within a torpor bout (early torpor, TE n = 5) and after 10–12 days in a torpor bout (late torpor, TL; n = 5). Animals that naturally rewarmed from prolonged torpor during an interbout arousal were sampled 2–3 h (early arousal AE; n = 7) and 10–12 h (late arousal AL; n = 5) after core body temperature increased above 30°C as indicated by the radiotransmitter. For sampling, summer active and naturally aroused animals were anesthetized with isoflurane, euthanized with sodium pentobarbital and decapitated. Torpid animals were euthanized by decapitation after verification of core body temperatures <7°C. Brains were removed from the skull, rapidly dissected, frozen in liquid nitrogen, and stored at −80°C.

#### Syrian hamsters (*Mesocricetus auratus*)

Syrian hamsters purchased from Harlan Winkelmann GmbH (Borchen, Germany) were bred and housed at the Medizinisch-Experimentelles Zentrum of the Medical Faculty of the University of Leipzig. In total, 41 animals were included in the study and subjected to hibernation conditions [47], [83]. The animals had free access to food and water and were maintained on an artificial 12∶12 h light dark cycle under conditions of constant temperature (22°C) and humidity. Briefly, they were transferred to the animal facility of the Paul Flechsig Institute, held at 23–26°C and an 8∶16 h light dark cycle for four to eight weeks and subsequently transferred to a chamber were they were maintained at 5–7°C with an 4∶20 h light dark cycle. General locomotor activity was monitored with custom build infrared detectors mounted on top of each cage allowing the discrimination between euthermic phases and torpor. Hibernating animals show torpor phases with inactivity of >24 h whereas non-hibernating euthermic animals did not. The status of the animals was confirmed by body temperature measurements (rectal), ranging between ∼7°C for hibernating animals and ∼34°C for euthermic hamsters (EU; n = 5 for Western blot and n = 2 for immunohistochemical analysis). For sampling, animals that have shown torpor at least three times were designated depending on their time of inactivity after an arousal episode as early torpor (TE; 8 h of inactivity; n = 5 for Western blot and n = 2 for immunohistochemistry) or late torpor (TL; 36–48 h of inactivity; n = 5 for Western blot and n = 3 for immunohistochemistry). According to the time after induction of arousal from a prolonged torpor period, animal groups were as designated early arousal (AE; 2.5 h; n = 5 for Western blot and n = 3 for immunohistochemistry) and late arousal (AL; 24–36 h; n = 5 for Western blot and n = 2 for immunohistochemistry). The immunohistochemical analysis of tau phosphorylation involved the study of two additional groups: torpid animals as characterised above but sampled after inactivity of 4 h (n = 2) as well as aroused animals as specified before but sampled 1 h after induced arousal (n = 2). For Western blot analysis animals were killed by CO_2_ ventilation and decapitated. Subsequently, the brain was removed, dissected, immediately frozen in liquid nitrogen and stored at −80°C.

#### American Black bears (*Ursus americanus*)

American black bears (31–118 kg) were captured May-July from the field either near McGrath or Anchorage, Alaska. Bears were transferred to Fairbanks, Alaska where they were held individually in a shaded outdoor holding facility. In summer and fall, bears were fed dog chow and had continuous access to water. Bears representing the non-hibernating condition were still feeding and active when they were euthanized and sampled for tissues between late May and late July (n = 6). Food was withdrawn 24 hours before these animals were sacrificed. Bears in the hibernating condition were euthanised for tissue sampling between March 1^st^-27^th^ (n = 5). These animals were without food since October 27^th^ and had been offered a diminishing food ration for two weeks prior. For monitoring of physiological conditions, bears used in hibernation experiments were surgically implanted in late November with telemetry transmitters for core body temperature (model TM, 27 MHz, Minimitter Inc., Sunriver, OR) and transmitters for ECG and EMG monitoring (models TL10M4-D70-CCP or TL10M4-D70-EEET, Data Sciences International, St. Paul, MI, USA or model T28F-14B, Konigsberg Instruments Inc., Pasadena, CA, USA) which transmitted both body core temperature and biopotentials. All transmitters were implanted intra-peritoneally. Beginning in late November, bears were housed in individual outdoor enclosures on the University of Alaska Fairbanks campus that had dens and straw material for nests. Dens were double walled boxes made from 2.5 cm thick plywood or high-density polyethylene with inside dimensions of approximately 0.8×1.0×1.0 m with 5 cm of Styrofoam between walls. Each nest box had a 46×46 cm door opening that was fitted with a double-layered breakaway door sealed by weather stripping and kept in place by metal clamps. The dens were instrumented with antennas and a thermocouple to monitor temperature. Air was continuously drawn from an exit hole at back of the den and oxygen consumption monitored as described previously [84]. On the day of tissue harvesting, bears were immobilized between 9:30–15:00 using Telazol (8–10 mg/kg) administered via pole syringe using a 16 g 1.5 inch needle or a CO_2_ powered pistol to administer Telazol via a standard Palmer dart. Immobilized bears were transported to a necropsy suite in a nearby building. After blood sampling, bears were euthanised by an intravenous injection of pentobarbital and death by cardiac arrest assessed with a stethoscope. Tissue collection followed immediately with samples frozen in liquid nitrogen and was complete within 15 min.

### Analysis of tau phosphorylation and tau expression

#### Immunohistochemistry

Animals were anesthetized with carbon dioxide and perfused transcardialy with physiological saline supplemented Heparin and afterwards with 4% paraformaldehyde and 0.1% glutaraldehyde in phosphate buffered saline (PBS). Subsequently the brain was removed, postfixed for 24 h in 4% paraformaldehyde in PBS, and cryoprotected by immersion in 30% sucrose in PBS for 48 h. The brains where cut into 30 µm thick sagittal sections and stored at 4°C in PBS with 0.01% sodium azide added as a preservative. Briefly, free-floating sections were processed for immunoperoxidase labelling: sections were rinsed in PBS, and incubated 30 min in 0.3% hydrogen peroxide in PBS. Sections were rinsed in TBS-T (TBS, 0.01% Triton X-100) and incubated for 60 min in 5% normal donkey serum (GE Healthcare, Freiburg, Germany; diluted in TBS-T). The primary antibody (AT8, Pierce; diluted 1∶2000 in TBS, 0.1% Triton X-100) was applied overnight. Subsequently, sections were rinsed, incubated 2 h with biotinylated donkey-anti-mouse (Dianova, Hamburg, Germany; diluted 1∶1000 in TBS, 0.1% Triton X-100), rinsed, incubated for 45 min with ExtrAvidin (Sigma, Taufkirchen, Germany; diluted 1∶1000 in TBS, 0.1% Triton X-100), rinsed, and developed with nickel-enhanced diaminobenzidine. Afterwards the sections were mounted, dehydrated, cleared, and coverslipped.

#### Protein extraction and Western blot

Brain tissue samples were ground in liquid nitrogen, aliquoted and stored at −80°C. Tissue samples for Western blot analysis were homogenised in 9 volumes (w/v) protein extraction buffer B (20 mM Tris-HCl, pH 7.2; 150 mM NaCl; 2 mM MgCl_2_; 2 mM EDTA; 2 mM EGTA; 1% NP40; 5 mM NaF; 1 mM Na_3_VO_4_; 5% glycerol; 1 mM PMSF; 1 µg/ml leupeptin; Complete protease inhibitor cocktail [Roche Diagnostics GmbH, Mannheim, Germany]) using Ultra Turrax. Homogenates were centrifuged (50,000×g, 30 min, 4°C), and supernatants were transferred into new tubes.

Protein concentration was determined using Bradford assay [85] and all samples were adjusted to equal protein concentration. SDS-PAGE was performed applying 20 µg total protein per lane. Proteins were separated on SDS-Polyacrylamide gels and then transferred to a PVDF or nitrocellulose membrane. Blots were blocked in blocking buffer (TBS; 2% BSA 0.05%; Tween 20) and thereafter probed with primary antibody (see Table 3) diluted in blocking buffer. Subsequently, blots were washed with TBS containing 0.05% Tween 20 and incubated with the corresponding secondary antibody (see Table 3) diluted in blocking buffer. Immunoreactivity was determined by enhanced chemiluminescence (0.23 mg/ml luminol; 0.1 mg/ml p-coumaric acid and 0.6 mg/ml sodium perborate in 0.1 M Tris-HCl, pH 8.6) and acquired with Kodak Image Station 2000R or with Hyperfilm ECL [GE Healthcare]. Blots were stripped (0.2 M glycine, pH 2.1; 1% Tween 20; 0.1% SDS) two hours at room temperature.

**Table 3:** List of applied antibodies.

Antigen	host	Dilution	Source/Designation
*Primary antibodies*			
anti-human tau	rabbit	1∶2000	Dako; A0024
AT270 [pT181]	mouse	1∶500	Pierce; MN1050
CP13 [pS202]	mouse	1∶500	Peter Davies
AT8 [pS202/pT205]	mouse	1∶500	Pierce; MN1020
AT100 [pT212/pS214/pT217]	mouse	1∶500	Pierce; MN1060
AT180 [pT231/pS235]	mouse	1∶500	Pierce; MN1040
p-tau - PHF13 [pS396]	mouse	1∶500	Santa Cruz; sc-32275
PHF1 [pS396/pS404]	mouse	1∶2000	Peter Davies
Alz-50 [conformation]	mouse	1∶300	Peter Davies
p-GSK3β [pS9]	rabbit	1∶1000	Cell Signaling; 9336S
GSK3β	mouse	1∶2000	BD Transduction; 610202
p-SAPK/JNK [pT183/pT185]	rabbit	1∶1000	Cell Signaling; 4668S
SAPK/JNK	rabbit	1∶1000	Cell Signaling; 9252
p-MAPK [pT183/pT185]	rabbit	1∶2000	Promega; V803A
ERK1 (C-16)	rabbit	1∶2000	Santa Cruz; sc-93
p-cdk5 [pS159]	rabbit	1∶500	Santa Cruz; sc-12919-R
cdk5 (C-8)	rabbit	1∶500	Santa Cruz; sc-172
Anti-β-Actin	mouse	1∶20,000	Sigma-Aldrich; A5316
*HRP-conjugated secondary antibodies*			
Mouse IgG	sheep	1∶10,000	GE Healthcare; NA931V
Rabbit IgG	donkey	1∶10,000	GE Healthcare; NA934V

The optical density of immunoreactivity was analysed based on 16-bit grey-level TIF-files using the 1D module of TotalLab software (Nonlinear Dynamics, Newcastle upon Tyne, UK). The immunoreactivity of phosphosite-specific tau antibodies was related to the expression of tau determined using an antibody directed against the non-phosphorylated protein. The uniformity of sample loading was confirmed by immunodetection of beta-actin and Coomassie-stain of the corresponding membrane after stripping.

### Analysis of tau-isoform expression

A potential, hibernation-state related alternative splicing of the tau exon 10 was determined in arctic ground squirrels (EU, n = 4; TL, n = 4, AL, n = 4). For this purpose neocortical brain tissue samples (70–100 mg) were homogenised in 1 ml Trizol reagent (Invitrogen, Karlsruhe, Germany) and mRNA was isolated according to the instructions of the product supplier. Quality and integrity of isolated mRNA was assessed by electrophoresis. Prior to the expression analysis a RT-PCR using degenerated primer (Pan-tau_F and Pan-tau_R; see Table 4) was performed to clone the Tau gene transcripts using one randomly selected sample. PCR products were isolated and ligated into PCR®2.1-TOPO vector (Invitrogen). The generated plasmid DNA was used for transformation of Mach-1 cells (Invitrogen) and isolated from selected clones. Based on the determined sequence (GenBank accession number: FJ609677) species-specific primers were designed (AGS-tau_TE10-F and AGS-tau_TE10-R; see Table 4). 1 µg of total RNA per sample was applied to generate first strand cDNA using Superscript reverse transcriptase (Invitrogen). 5% of the reaction volume was subsequently used for PCR and the products were separated in 1.5% agarose gels. The optical density of the expressed tau isoforms was analysed based on 8-bit grey-level TIF-files using the 1D module of TotalLab software (Nonlinear Dynamics) and calculated as percentage related to the summative intensity of all bands (set to 100%).

**Table 4:** List of applied primers for cloning and isoforms expression analysis of tau in arctic ground squirrels.

Primer designation	Primer sequence (5′ 3′)
Pan-tau_F	GGC TAC AGC AGC CCC GGC TC
Pan-tau_R	TGA TCA CAA ACC CTG CTT GG
AGS-tau_TE10-F	GAA CGT CAG GTC CAA GAT CG
AGS-tau_TE10-R	CTT GGC TTT GGC ATT CTC TC

### Phospho-protein staining

To quantify the state-dependent formation of phospho-proteins neocortical brain extracts of arctic ground squirrels (EU, n = 4; TL, n = 4, AL, n = 4) were separated by SDS-PAGE and transferred to a PVDF membrane (see 2.2.1). The proteins were stained using the Pro-Q Diamond phosphoprotein blot stain kit (Invitrogen, Karlsruhe, Germany) according to the instructions of the product supplier.

### Analysis of temperature dependent tau phosphate net turnover

To determine the temperature sensitivity of enzyme activity we used cortical brain samples from arctic ground squirrels (EU, n = 4; TL, n = 4, AL, n = 4) that were homogenized with an Ultra Turrax in five volumes of buffer K (20 mM Tris-HCl, pH 7.2; 150 mM NaCl; 0.5% NP40; 0.25% sodium deoxycholate; 5% glycerol; 1 mM AEBSF and Complete protease inhibitor cocktail [Roche]). Protein concentrations were determined according to Bradford [85]. 12 µg total protein were added to a total of 50 µl assay buffer (50 mM Hepes, pH 7.4; 10 mM MgCl_2_; 10 mM MnCl_2_; 80 ng/µl recombinant tau protein; 100 µm ATP [cold]; 50 nCi/µl ^32^P ATP) and incubated at various temperatures and times. Reactions for the analysis of tau phosphate net turnover were started at 37°C and aliquots were taken successively after incubation at temperatures that were lowered stepwise. To determine the tau phosphate net turnover in an increasing temperature gradient the pre-phosphorylation of tau was performed by 15 min incubation at 30°C and samples were immediately chilled on ice. The temperature was increased stepwise and aliquots were taken successively from the samples after incubation. The record of reaction was started after incubation at 5°C. Reactions were terminated by adding SDS-sample buffer followed by heat denaturation at 95°C for 10 min. Samples were separated by SDS-PAGE; gels were dried and exposed to X-ray film. Optical density was analysed using the 1D module of TotalLab software (Nonlinear Dynamics) based on 16-bit grey-level film scans.

### Analysis of the activity state profile of tau kinases in hibernating animals

The activity state of the tau kinases glycogen synthase kinase 3 beta (GSK3-beta), cyclin dependent kinase 5 (cdk5), stress-activated protein kinase/Jun-amino-terminal kinase (SAPK/JNK) and mitogen activated protein kinase/extracellular regulated protein kinase (MAPK/ERK), was determined by Western blot as described previously (2.2.1). Immunoreactivities of the phosphosite-specific antibodies were related to the expression of the according proteins determined applying antibodies directed against the non-modified molecule (Table 3). The consistency of sample loading was verified by the immunodetection of beta-actin and Coomassie-stain of the blot membrane subsequent to stripping.

## Supporting Information

Table S1 Summary of hibernation-dependent tau phosphorylation in arctic ground squirrels (*Spermophilus parryii*) and Syrian hamsters (*Mesocricetus auratus*). The table lists the increase of tau phosphorylation at specific sites in different brain regions during the hibernation cycle (TE - early torpor; TL - late torpor; AE - early arousal; AL - late arousal). Data are based on Western blot experiments. Increase factors are related to the level of phosphorylation in euthermic animals. One way ANOVA (unblocked) was performed and italic p-values indicate significant alterations (p≤0.05). Newman-Keuls contrasts are listed to indicate the difference in tau phosphorylation level between all analysed groups.
(0.04 MB DOC)

## References

[1] Binder LI, Frankfurter A, Rebhun LI (1985). The distribution of tau in the mammalian central nervous system.. J Cell Biol.

[2] Goedert M, Spillantini MG, Jakes R, Rutherford D, Crowther RA (1989). Multiple isoforms of human microtubule-associated protein tau: sequences and localization in neurofibrillary tangles of Alzheimer's disease.. Neuron.

[3] Janke C, Beck M, Stahl T, Holzer M, Brauer K (1999). Phylogenetic diversity of the expression of the microtubule-associated protein tau: implications for neurodegenerative disorders.. Brain Res Mol Brain Res.

[4] Bullmann T, de Silva R, Holzer M, Mori H, Arendt T (2007). Expression of embryonic tau protein isoforms persist during adult neurogenesis in the hippocampus.. Hippocampus.

[5] Weingarten MD, Lockwood AH, Hwo SY, Kirschner MW (1975). A protein factor essential for microtubule assembly.. Proc Natl Acad Sci USA.

[6] Cleveland DW, Hwo SY, Kirschner MW (1977). Physical and chemical properties of purified tau factor and the role of tau in microtubule assembly.. J Mol Biol.

[7] Samsonov A, Yu JZ, Rasenick M, Popov SV (2004). Tau interaction with microtubules in vivo.. J Cell Sci.

[8] Dayanandan R, Van SM, Mack TG, Ko L, Yen SH (1999). Mutations in tau reduce its microtubule binding properties in intact cells and affect its phosphorylation.. FEBS Lett.

[9] Goedert M, Spillantini MG, Cairns NJ, Crowther RA (1992). Tau proteins of Alzheimer paired helical filaments: abnormal phosphorylation of all six brain isoforms.. Neuron.

[10] Bramblett GT, Goedert M, Jakes R, Merrick SE, Trojanowski JQ (1993). Abnormal tau phosphorylation at Ser396 in Alzheimer's disease recapitulates development and contributes to reduced microtubule binding.. Neuron.

[11] Biernat J, Gustke N, Drewes G, Mandelkow EM, Mandelkow E (1993). Phosphorylation of Ser262 strongly reduces binding of tau to microtubules: distinction between PHF-like immunoreactivity and microtubule binding.. Neuron.

[12] Illenberger S, Zheng-Fischhofer Q, Preuss U, Stamer K, Baumann K (1998). The endogenous and cell cycle-dependent phosphorylation of tau protein in living cells: implications for Alzheimer's disease.. Mol Biol Cell.

[13] Williams DR (2006). Tauopathies: classification and clinical update on neurodegenerative diseases associated with microtubule-associated protein tau.. Intern Med J.

[14] Grundke-Iqbal I, Iqbal K, Tung YC, Quinlan M, Wisniewski HM (1986). Abnormal phosphorylation of the microtubule-associated protein tau (tau) in Alzheimer cytoskeletal pathology.. Proc Natl Acad Sci USA.

[15] Bielska AA, Zondlo NJ (2006). Hyperphosphorylation of Tau Induces Local Polyproline II Helix.. Biochemistry.

[16] Du JT, Yu CH, Zhou LX, Wu WH, Lei P (2007). Phosphorylation modulates the local conformation and self-aggregation ability of a peptide from the fourth tau microtubule-binding repeat.. FEBS J.

[17] Alonso AC, Grundke-Iqbal I, Iqbal K (1996). Alzheimer's disease hyperphosphorylated tau sequesters normal tau into tangles of filaments and disassembles microtubules.. Nat Med.

[18] Alonso A, Zaidi T, Novak M, Grundke-Iqbal I, Iqbal K (2001). Hyperphosphorylation induces self-assembly of tau into tangles of paired helical filaments/straight filaments.. Proc Natl Acad Sci USA.

[19] Chohan MO, Haque N, Alonso A, El AE, Grundke-Iqbal I (2005). Hyperphosphorylation-induced self assembly of murine tau: a comparison with human tau.. J Neural Transm.

[20] Schneider A, Biernat J, von Bergen M, Mandelkow E, Mandelkow EM (1999). Phosphorylation that detaches tau protein from microtubules (Ser262, Ser214) also protects it against aggregation into Alzheimer paired helical filaments.. Biochemistry.

[21] Haase C, Stieler JT, Arendt T, Holzer M (2004). Pseudophosphorylation of tau protein alters its ability for self-aggregation.. J Neurochem.

[22] Ding H, Matthews TA, Johnson GV (2006). Site-specific phosphorylation and caspase cleavage differentially impact tau-microtubule interactions and tau aggregation.. J Biol Chem.

[23] Liu F, Li B, Tung EJ, Grundke-Iqbal I, Iqbal K (2007). Site-specific effects of tau phosphorylation on its microtubule assembly activity and self-aggregation.. Eur J Neurosci.

[24] Arendt T, Stieler J, Strijkstra AM, Hut RA, Rüdiger J (2003). Reversible paired helical filament-like phosphorylation of tau is an adaptive process associated with neuronal plasticity in hibernating animals.. J Neurosci.

[25] King ME (2005). Can tau filaments be both physiologically beneficial and toxic?. Biochim Biophys Acta.

[26] Lee HG, Perry G, Moreira PI, Garrett MR, Liu Q (2005). Tau phosphorylation in Alzheimer's disease: pathogen or protector?. Trends Mol Med.

[27] Härtig W, Stieler J, Boerema AS, Wolf J, Schmidt U (2007). Hibernation model of tau phosphorylation in hamsters: selective vulnerability of cholinergic basal forebrain neurons - implications for Alzheimer's disease.. Eur J Neurosci.

[28] Stieler JT, Boerema AS, Bullmann T, Kohl F, Strijkstra AM, Lovegrove BG, McKechnie AE (2008). Activity state profile of tau kinases in hibernating animals.. Hypometabolism in animals: hibernation, torpor and cryobiology.

[29] Su B, Wang X, Drew KL, Perry G, Smith MA (2008). Physiological regulation of tau phosphorylation during hibernation.. J Neurochem.

[30] Popov VI, Bocharova LS (1992). Hibernation-induced structural changes in synaptic contacts between mossy fibres and hippocampal pyramidal neurons.. Neuroscience.

[31] Strijkstra AM, Hut RA, de Wilde MC, Stieler J, Van der Zee EA (2003). Hippocampal synaptophysin immunoreactivity is reduced during natural hypothermia in ground squirrels.. Neurosci Lett.

[32] von der Ohe CG, Darian-Smith C, Garner CC, Heller HC (2006). Ubiquitous and temperature-dependent neural plasticity in hibernators.. J Neurosci.

[33] von der Ohe CG, Garner CC, Darian-Smith C, Heller HC (2007). Synaptic protein dynamics in hibernation.. J Neurosci.

[34] Millesi E, Prossinger H, Dittami JP, Fieder M (2001). Hibernation effects on memory in European ground squirrels (Spermophilus citellus).. J Biol Rhythms.

[35] Jicha GA, Bowser R, Kazam IG, Davies P (1997). Alz-50 and MC-1, a new monoclonal antibody raised to paired helical filaments, recognize conformational epitopes on recombinant tau.. J Neurosci Res.

[36] Cross DA, Alessi DR, Cohen P, Andjelkovich M, Hemmings BA (1995). Inhibition of glycogen synthase kinase-3 by insulin mediated by protein kinase B.. Nature.

[37] Heldmaier G, Ortmann S, Elvert R (2004). Natural hypometabolism during hibernation and daily torpor in mammals.. Respir Physiol Neurobiol.

[38] Barnes BM (1989). Freeze avoidance in a mammal: body temperatures below 0 degree C in an Arctic hibernator.. Science.

[39] Dausmann KH, Glos J, Ganzhorn JU, Heldmaier G (2005). Hibernation in the tropics: lessons from a primate.. J Comp Physiol [B].

[40] Daan S, Barnes BM, Strijkstra AM (1991). Warming up for sleep? Ground squirrels sleep during arousals from hibernation.. Neurosci Lett.

[41] Srere HK, Wang LC, Martin SL (1992). Central role for differential gene expression in mammalian hibernation.. Proc Natl Acad Sci USA.

[42] Grigg GC, Beard LA, Augee ML (2004). The evolution of endothermy and its diversity in mammals and birds.. Physiol Biochem Zool.

[43] Buck CL, Barnes BM (2000). Effects of ambient temperature on metabolic rate, respiratory quotient, and torpor in an arctic hibernator.. Am J Physiol Regul Integr Comp Physiol.

[44] Nelson RA (1973). Winter sleep in the black bear. A physiologic and metabolic marvel.. Mayo Clin Proc.

[45] Nelson RA, Jones JD, Wahner HW (1975). Nitrogen metabolism in bears: urea metabolism in summer starvation and in winter sleep and role of urinary bladder in water and nitrogen conservation.. Mayo Clin Proc.

[46] Tøien Ø, Barnes BM, Blake J, Grahn D, Heller HC (1999). Hibernation in black bears: Energetics and thermoregulation.. FASEB J.

[47] Oklejewicz M, Daan S, Strijkstra AM (2001). Temporal organisation of hibernation in wild-type and tau mutant Syrian hamsters.. J Comp Physiol [B].

[48] Lyman CP (1948). The oxygen consumption and temperature regulation of hibernating hamsters.. J Exp Zool.

[49] Yanagisawa M, Planel E, Ishiguro K, Fujita SC (1999). Starvation induces tau hyperphosphorylation in mouse brain: implications for Alzheimer's disease.. FEBS Lett.

[50] Planel E, Yasutake K, Fujita SC, Ishiguro K (2001). Inhibition of protein phosphatase 2A overrides tau protein kinase I/glycogen synthase kinase 3 beta and cyclin-dependent kinase 5 inhibition and results in tau hyperphosphorylation in the hippocampus of starved mouse.. J Biol Chem.

[51] Planel E, Miyasaka T, Launey T, Chui DH, Tanemura K (2004). Alterations in glucose metabolism induce hypothermia leading to tau hyperphosphorylation through differential inhibition of kinase and phosphatase activities: implications for Alzheimer's disease.. J Neurosci.

[52] Ikeda Y, Ishiguro K, Fujita SC (2007). Ether stress-induced Alzheimer-like tau phosphorylation in the normal mouse brain.. FEBS Lett.

[53] Planel E, Richter KE, Nolan CE, Finley JE, Liu L (2007). Anesthesia leads to tau hyperphosphorylation through inhibition of phosphatase activity by hypothermia.. J Neurosci.

[54] Okawa Y, Ishiguro K, Fujita SC (2003). Stress-induced hyperphosphorylation of tau in the mouse brain.. FEBS Lett.

[55] Wang JZ, Liu F (2008). Microtubule-associated protein tau in development, degeneration and protection of neurons.. Prog Neurobiol.

[56] Gärtner U, Janke C, Holzer M, Vanmechelen E, Arendt T (1998). Postmortem changes in the phosphorylation state of tau-protein in the rat brain.. Neurobiol Aging.

[57] Mercken M, Vandermeeren M, Lübke U, Six J, Boons J (1992). Monoclonal antibodies with selective specificity for Alzheimer Tau are directed against phosphatase-sensitive epitopes.. Acta Neuropathol.

[58] Goedert M, Jakes R, Crowther RA, Cohen P, Vanmechelen E (1994). Epitope mapping of monoclonal antibodies to the paired helical filaments of Alzheimer's disease: identification of phosphorylation sites in tau protein.. Biochem J.

[59] Yoshida H, Goedert M (2006). Sequential phosphorylation of tau protein by cAMP-dependent protein kinase and SAPK4/p38delta or JNK2 in the presence of heparin generates the AT100 epitope.. J Neurochem.

[60] Plattner F, Angelo M, Giese KP (2006). The roles of cyclin-dependent kinase 5 and glycogen synthase kinase 3 in tau hyperphosphorylation.. J Biol Chem.

[61] Sharma P, Sharma M, Amin ND, Albers RW, Pant HC (1999). Regulation of cyclin-dependent kinase 5 catalytic activity by phosphorylation.. Proc Natl Acad Sci USA.

[62] Stieler JT, Bullmann T, Kohl F, Barnes BM, Arendt T (2009). PHF-like tau phosphorylation in mammalian hibernation is not associated with p25-formation.. J Neural Transm.

[63] Zhu X, Smith MA, Perry G, Wang Y, Ross AP (2005). MAPKs are differentially modulated in arctic ground squirrels during hibernation.. J Neurosci Res.

[64] MacDonald JA, Storey KB (2005). Mitogen-activated protein kinases and selected downstream targets display organ-specific responses in the hibernating ground squirrel.. Int J Biochem Cell Biol.

[65] Reiman EM, Caselli RJ, Yun LS, Chen K, Bandy D (1996). Preclinical evidence of Alzheimer's disease in persons homozygous for the epsilon 4 allele for apolipoprotein E.. N Engl J Med.

[66] Small GW, Ercoli LM, Silverman DH, Huang SC, Komo S (2000). Cerebral metabolic and cognitive decline in persons at genetic risk for Alzheimer's disease.. Proc Natl Acad Sci USA.

[67] Reiman EM, Chen K, Alexander GE, Caselli RJ, Bandy D (2004). Functional brain abnormalities in young adults at genetic risk for late-onset Alzheimer's dementia.. Proc Natl Acad Sci USA.

[68] de Leon MJ, Convit A, Wolf OT, Tarshish CY, DeSanti S (2001). Prediction of cognitive decline in normal elderly subjects with 2-[(18)F]fluoro-2-deoxy-D-glucose/poitron-emission tomography (FDG/PET).. Proc Natl Acad Sci USA.

[69] Devous MDS (2002). Functional brain imaging in the dementias: role in early detection, differential diagnosis, and longitudinal studies.. Eur J Nucl Med Mol Imaging.

[70] Kim YK, Lee DS, Lee SK, Kim SK, Chung CK (2003). Differential features of metabolic abnormalities between medial and lateral temporal lobe epilepsy: quantitative analysis of (18)F-FDG PET using SPM.. J Nucl Med.

[71] Swaab DF, Dubelaar EJ, Scherder EJ, van Someren EJ, Verwer RW (2003). Therapeutic strategies for Alzheimer disease: focus on neuronal reactivation of metabolically impaired neurons.. Alzheimer Dis Assoc Disord.

[72] Kalmijn S, Mehta KM, Pols HA, Hofman A, Drexhage HA (2000). Subclinical hyperthyroidism and the risk of dementia. The Rotterdam study.. Clin Endocrinol (Oxf).

[73] Schubert M, Brazil DP, Burks DJ, Kushner JA, Ye J (2003). Insulin receptor substrate-2 deficiency impairs brain growth and promotes tau phosphorylation.. J Neurosci.

[74] Schubert M, Gautam D, Surjo D, Ueki K, Baudler S (2004). Role for neuronal insulin resistance in neurodegenerative diseases.. Proc Natl Acad Sci USA.

[75] Iqbal K, Grundke-Iqbal I (2005). Metabolic/signal transduction hypothesis of Alzheimer's disease and other tauopathies.. Acta Neuropathol.

[76] Hoyer S (1998). Is sporadic Alzheimer disease the brain type of non-insulin dependent diabetes mellitus? A challenging hypothesis.. J Neural Transm.

[77] Gasparini L, Xu H (2003). Potential roles of insulin and IGF-1 in Alzheimer's disease.. Trends Neurosci.

[78] Haley AP, Knight-Scott J, Simnad VI, Manning CA (2006). Increased glucose concentration in the hippocampus in early Alzheimer's disease following oral glucose ingestion.. Magn Reson Imaging.

[79] Heininger K (2000). A unifying hypothesis of Alzheimer's disease. IV. Causation and sequence of events.. Rev Neurosci.

[80] Friedhoff P, Schneider A, Mandelkow EM, Mandelkow E (1998). Rapid assembly of Alzheimer-like paired helical filaments from microtubule-associated protein tau monitored by fluorescence in solution.. Biochemistry.

[81] Cork LC, Powers RE, Selkoe DJ, Davies P, Geyer JJ (1988). Neurofibrillary tangles and senile plaques in aged bears.. J Neuropathol Exp Neurol.

[82] Long RA, Hut RA, Barnes BM (2007). Simultaneous Collection of Body Temperature and Activity Data in Burrowing Mammals: a New Technique.. J Wildl Manage.

[83] Ueda S, Ibuka N (1995). An analysis of factors that induce hibernation in Syrian hamsters.. Physiol Behav.

[84] Fedorov VB, Goropashnaya AV, Toien O, Stewart NC, Gracey AY (2009). Elevated expression of protein biosynthesis genes in liver and muscle of hibernating black bears (Ursus americanus).. Physiol Genomics.

[85] Bradford MM (1976). A rapid and sensitive method for the quantitation of microgram quantities of protein utilizing the principle of protein dye binding.. Anal Biochem.

