# Supplementary material for: The Physiological Link between Metabolic Rate Depression and Tau Phosphorylation in Mammalian Hibernation
Source: PLoS One. 2011 Jan 18;6(1):e14530. doi: 10.1371/journal.pone.0014530 (PMC3022585; doi:10.1371/journal.pone.0014530)
Supplement: Table S1 — Summary of hibernation-dependent tau phosphorylation in arctic ground squirrels (Spermophilus parryii) and Syrian hamsters (Mesocricetus auratus). The table lists the increase of tau phosphorylation at specific sites in different brain regions during the hibernation cycle (TE - early torpor; TL - late torpor; AE - early arousal; AL - late arousal). Data are based on Western blot experiments. Increase factors are related to the level of phosphorylation in euthermic animals. One way ANOVA (unblocked) was performed and italic p-values indicate significant alterations (p≤0.05). Newman-Keuls contrasts are listed to indicate the difference in tau phosphorylation level between all analysed groups. (0.04 MB DOC) [file pone.0014530.s001.doc]

| ***Mesocricetus auratus*** | | | | | | | |
| --- | --- | --- | --- | --- | --- | --- | --- |
| **phospho site** | **Brain region** | **Increase factor** | | | | **p value**  **(ANOVA)** | **Newman-Keuls Contrasts** |
|  |  | **TE** | **TL** | **AE** | **AL** |  |  |
| total tau | Neocortex | 1.05 | 1.01 | 1.07 | 1.09 | 0.4960 |  |
|  | Hippocampus | 1.08 | 1.10 | 1.09 | 1.08 | 0.8020 |  |
|  | Cerebellum | 0.96 | 0.87 | 0.98 | 0.89 | *0.0110* | EU vs. TL (p<0.05) |
|  | Brainstem | 1.06 | 1.14 | 1.18 | 1.08 | 0.0542 |  |
|  | Midbrain | 1.03 | 0.95 | 1.11 | 1.01 | 0.0753 |  |
| T181 | Neocortex | 1.29 | 1.30 | 1.00 | 0.98 | *0.0007* | EU vs. TE (p<0.01) EU vs. TL (p<0.05) TE vs. AE (p<0.01) TE vs. AL (p<0.01) TL vs. AE (p<0.01) TL vs. AL (p<0.01) |
|  | Hippocampus | 1.52 | 1.55 | 1.31 | 1.26 | *0.0041* | EU vs. TE (p<0.01) EU vs. TL (p<0.01) |
|  | Cerebellum | 1.17 | 1.23 | 1.04 | 1.10 | 0.0710 |  |
|  | Brainstem | 1.20 | 1.20 | 0.96 | 0.98 | 0.8378 |  |
|  | Midbrain | 1.34 | 1.37 | 1.07 | 0.93 | *0.0062* | TE vs. AL (p<0.05) TL vs. AL (p<0.05) |
| S202 | Neocortex | 1.38 | 1.32 | 1.03 | 0.98 | 0.0740 |  |
|  | Hippocampus | 1.23 | 1.13 | 0.68 | 0.76 | 0.1205 |  |
|  | Cerebellum | 1.22 | 1.24 | 0.76 | 0.72 | *0.0044* | TE vs. AE (p<0.05) TE vs. AL (p<0.05) TL vs. AE (p<0.05) TL vs. AL (p<0.05) |
|  | Brainstem | 1.04 | 0.81 | 0.83 | 0.98 | 0.7086 |  |
|  | Midbrain | 1.21 | 0.96 | 0.96 | 0.87 | 0.1769 |  |
| S202/ T205 | Neocortex | 1.93 | 1.92 | 1.06 | 1.04 | *0.0001* | EU vs. TE (p<0.01) EU vs. TL (p<0.01) TE vs. AE (p<0.01) TE vs. AL (p<0.01) TL vs. AE (p<0.01) TL vs. AL (p<0.01) |
|  | Hippocampus | 1.10 | 1.13 | 0.66 | 0.57 | 0.2200 |  |
|  | Cerebellum | 1.62 | 1.47 | 0.72 | 0.90 | *0.0019* | EU vs. TE (p<0.05) EU vs. TL (p<0.05) TE vs. AE (p<0.01) TE vs. AL (p<0.01) TL vs. AE (p<0.05) TL vs. AL (p<0.05) |
|  | Brainstem | 1.22 | 0.92 | 1.13 | 1.08 | 0.8340 |  |
|  | Midbrain | 2.52 | 2.52 | 1.20 | 1.03 | *0.0001* | EU vs. TE (p<0.01) EU vs. TL (p<0.01) TE vs. AE (p<0.01) TE vs. AL (p<0.01) TL vs. AE (p<0.01) TL vs. AL (p<0.01) |
| T212/ S214/ T217 | Neocortex | 1.44 | 1.34 | 0.97 | 1.00 | *0.0007* | EU vs. TE (p<0.01) EU vs. TL (p<0.01) TE vs. AE (p<0.01) TE vs. AL (p<0.01) TL vs. AE (p<0.05) TL vs. AL (p<0.05) |
|  | Hippocampus | 1.50 | 1.44 | 1.17 | 0.85 | 0.1852 |  |
|  | Cerebellum | 1.23 | 1.55 | 1.15 | 0.99 | 0.4283 |  |
|  | Brainstem | 1.97 | 2.20 | 1.34 | 2.35 | 0.6893 |  |
|  | Midbrain | 2.68 | 2.91 | 1.64 | 1.65 | *0.0114* | EU vs. TE (p<0.05) EU vs. TL (p<0.05) |
| T231/ S235 | Neocortex | 3.68 | 5.06 | 1.51 | 1.25 | *0.0001* | EU vs. TE (p<0.01) EU vs. TL (p<0.01) TE vs. TL (p<0.05) TE vs. AE (p<0.01) TE vs. AL (p<0.01) TL vs. AE (p<0.01) TL vs. AL (p<0.01) |
|  | Hippocampus | 2.28 | 3.29 | 1.19 | 0.85 | *0.0001* | EU vs. TE (p<0.05) EU vs. TL (p<0.01) TE vs. TL (p<0.05) TE vs. AE (p<0.05) TE vs. AL (p<0.01) TL vs. AE (p<0.01) TL vs. AL (p<0.01) |
|  | Cerebellum | 4.72 | 5.99 | 1.80 | 1.50 | *0.0004* | EU vs. TE (p<0.05) EU vs. TL (p<0.01) TE vs. AE (p<0.05) TE vs. AL (p<0.05) TL vs. AE (p<0.01) TL vs. AL (p<0.01) |
|  | Brainstem | 1.65 | 2.45 | 1.47 | 0.84 | *0.0019* | EU vs. TL (p<0.01) TE vs. TL (p<0.05) TL vs. AE (p<0.05) TL vs. AL (p<0.01) |
|  | Midbrain | 2.81 | 3.62 | 1.59 | 0.82 | *0.0001* | EU vs. TE (p<0.01) EU vs. TL (p<0.01) TE vs. TL (p<0.05) TE vs. AE (p<0.01) TE vs. AL (p<0.01) TL vs. AE (p<0.01) TL vs. AL (p<0.01) |
| S396 | Neocortex | 1.56 | 1.74 | 0.63 | 0.88 | 0.2731 |  |
|  | Hippocampus | 2.47 | 2.58 | 2.91 | 1.21 | 0.0978 |  |
|  | Cerebellum | 0.62 | 0.55 | 0.63 | 0.87 | 0.0709 |  |
|  | Brainstem | 1.21 | 1.08 | 0.60 | 0.77 | 0.1551 |  |
|  | Midbrain | 1.47 | 1.57 | 0.85 | 0.98 | *0.0025* | EU vs. TE (p<0.05) EU vs. TL (p<0.05) TE vs. AE (p<0.05) TE vs. AL (p<0.05) TL vs. AE (p<0.05) TL vs. AL (p<0.05) |
| S396/ S404 | Neocortex | 1.38 | 1.31 | 0.90 | 0.89 | *0.0001* | EU vs. TE (p<0.01) EU vs. TL (p<0.01) TE vs. AE (p<0.01) TE vs. AL (p<0.01) TL vs. AE (p<0.01) TL vs. AL (p<0.01) |
|  | Hippocampus | 1.61 | 1.54 | 1.08 | 1.11 | *0.0006* | EU vs. TE (p<0.01) EU vs. TL (p<0.01) TE vs. AE (p<0.01) TE vs. AL (p<0.01) TL vs. AE (p<0.05) TL vs. AL (p<0.01) |
|  | Cerebellum | 1.40 | 1.46 | 0.97 | 1.03 | *0.0002* | EU vs. TE (p<0.01) EU vs. TL (p<0.01) TE vs. AE (p<0.01) TE vs. AL (p<0.01) TL vs. AE (p<0.01) TL vs. AL (p<0.01) |
|  | Brainstem | 1.18 | 1.18 | 0.90 | 0.92 | *0.0115* |  |
|  | Midbrain | 1.50 | 1.55 | 1.12 | 1.23 | 0.1389 |  |

| ***Spermophilus parryii*** | | | | | | | |
| --- | --- | --- | --- | --- | --- | --- | --- |
| **phospho site** | **Brain region** | **Increase factor** | | | | **p value**  **(ANOVA)** | **Newman-Keuls Contrasts** |
|  |  | **TE** | **TL** | **AE** | **AL** |  |  |
| total tau | Neocortex | 1.01 | 1.07 | 0.95 | 1.01 | 0.4124 |  |
|  | Hippocampus | 0.87 | 0.92 | 0.96 | 0.95 | 0.1592 |  |
|  | Cerebellum | 1.13 | 1.15 | 1.06 | 1.03 | 0.4731 |  |
|  | Brainstem | 0.83 | 0.83 | 0.89 | 0.95 | *0.0001* | EU vs. TE (p<0.01) EU vs. TL (p<0.01) EU vs. AE (p<0.01) TE vs. AL (p<0.05) TL vs. AL (p<0.05) |
|  | Midbrain | 0.76 | 0.82 | 0.88 | 0.89 | *0.0001* | EU vs. TE (p<0.01) EU vs. TL (p<0.01) EU vs. AE (p<0.01) EU vs. AL (p<0.01) TE vs. AE (p<0.01) TE vs. AL (p<0.05) |
| T181 | Neocortex | 1.39 | 1.07 | 1.25 | 0.78 | 0.1005 |  |
|  | Hippocampus | 1.05 | 1.15 | 1.14 | 0.89 | 0.8159 |  |
|  | Cerebellum | 1.00 | 1.09 | 1.01 | 0.96 | 0.9440 |  |
|  | Brainstem | 1.68 | 1.76 | 1.24 | 1.31 | *0.0054* | EU vs. TE (p<0.05) EU vs. TL (p<0.01) |
|  | Midbrain | 1.64 | 2.22 | 1.51 | 1.41 | *0.0001* | EU vs. TE (p<0.05) EU vs. TL (p<0.01) EU vs. AE (p<0.05) TE vs. TL (p<0.05) TL vs. AE (p<0.01) TL vs. AL (p<0.01) |
| S202 | Neocortex | 1.22 | 0.78 | 1.76 | 0.75 | 0.1002 |  |
|  | Hippocampus | 1.10 | 1.15 | 1.40 | 1.28 | 0.2664 |  |
|  | Cerebellum | 1.09 | 1.11 | 1.21 | 1.26 | 0.8072 |  |
|  | Brainstem | 1.44 | 0.97 | 1.32 | 1.08 | 0.1001 |  |
|  | Midbrain | 1.28 | 1.15 | 1.45 | 1.30 | 0.4275 |  |
| S202/ T205 | Neocortex | 2.02 | 1.68 | 1.55 | 1.17 | *0.0089* | EU vs. TE (p<0.05) |
|  | Hippocampus | 1.55 | 1.42 | 1.43 | 1.33 | 0.1796 |  |
|  | Cerebellum | 1.37 | 1.05 | 1.42 | 0.97 | 0.3137 |  |
|  | Brainstem | 1.71 | 1.30 | 1.30 | 1.14 | *0.0106* | EU vs. TE (p<0.01) TE vs. AL (p<0.05) |
|  | Midbrain | 1.87 | 1.71 | 1.56 | 1.38 | *0.0002* | EU vs. TE (p<0.01) EU vs. TL (p<0.01) EU vs. AE (p<0.01) EU vs. AL (p<0.05) |
| T212/ S214/ T217 | Neocortex | 1.95 | 1.18 | 1.60 | 0.98 | *0.0224* |  |
|  | Hippocampus | 1.45 | 1.26 | 1.32 | 1.28 | 0.3782 |  |
|  | Cerebellum | 0.89 | 0.88 | 1.07 | 1.05 | 0.7015 |  |
|  | Brainstem | 1.44 | 1.37 | 1.12 | 1.02 | 0.1299 |  |
|  | Midbrain | 1.85 | 1.74 | 1.21 | 1.11 | *0.0004* | EU vs. TE (p<0.01) EU vs. TL (p<0.01) TE vs. AE (p<0.05) TE vs. AL (p<0.05) TL vs. AE (p<0.05) TL vs. AL (p<0.05) |
| T231/ S235 | Neocortex | 2.81 | 3.46 | 1.52 | 1.14 | *0.0001* | EU vs. TE (p<0.01) EU vs. TL (p<0.01) EU vs. AE (p<0.05) TE vs. TL (p<0.05) TE vs. AE (p<0.01) TE vs. AL (p<0.01) TL vs. AE (p<0.01) TL vs. AL (p<0.01) |
|  | Hippocampus | 2.38 | 2.99 | 1.47 | 1.02 | *0.0001* | EU vs. TE (p<0.01) EU vs. TL (p<0.01) TE vs. AE (p<0.01) TE vs. AL (p<0.01) TL vs. AE (p<0.01) TL vs. AL (p<0.01) |
|  | Cerebellum | 1.78 | 2.84 | 1.46 | 1.09 | *0.0001* | EU vs. TE (p<0.05) EU vs. TL (p<0.01) TE vs. TL (p<0.01) TL vs. AE (p<0.01) TL vs. AL (p<0.01) |
|  | Brainstem | 1.75 | 2.05 | 1.31 | 1.04 | *0.0002* | EU vs. TE (p<0.01) EU vs. TL (p<0.01) TE vs. AL (p<0.05) TL vs. AE (p<0.01) TL vs. AL (p<0.01) |
|  | Midbrain | 2.99 | 4.40 | 1.68 | 1.23 | *0.0001* | EU vs. TE (p<0.01) EU vs. TL (p<0.01) EU vs. AE (p<0.05) TE vs. TL (p<0.01) TE vs. AE (p<0.01) TE vs. AL (p<0.01) TL vs. AE (p<0.01) TL vs. AL (p<0.01) |
| S396 | Neocortex | 1.62 | 1.75 | 1.37 | 1.02 | *0.0051* | EU vs. TE (p<0.05) EU vs. TL (p<0.05) TE vs. AL (p<0.05) TL vs. AE (p<0.05) |
|  | Hippocampus | 1.18 | 1.15 | 0.95 | 0.83 | 0.8978 |  |
|  | Cerebellum | 1.41 | 1.35 | 1.39 | 1.14 | 0.2388 |  |
|  | Brainstem | 1.81 | 1.94 | 1.47 | 1.45 | *0.0002* | EU vs. TE (p<0.01) EU vs. TL (p<0.01) EU vs. AE (p<0.05) EU vs. AL (p<0.05) |
|  | Midbrain | 1.54 | 2.00 | 1.78 | 1.52 | *0.0037* | EU vs. TL (p<0.01) EU vs. AE (p<0.01) |
| S396/ S404 | Neocortex | 1.75 | 1.37 | 1.35 | 1.08 | *0.0007* | EU vs. TE (p<0.01) TE vs. AL (p<0.01) |
|  | Hippocampus | 1.13 | 1.08 | 1.19 | 0.95 | 0.4571 |  |
|  | Cerebellum | 1.14 | 1.07 | 1.19 | 0.95 | 0.4744 |  |
|  | Brainstem | 1.84 | 1.66 | 1.41 | 1.32 | *0.0013* | EU vs. TE (p<0.01) EU vs. TL (p<0.01) |
|  | Midbrain | 2.10 | 2.37 | 1.70 | 1.48 | *0.0001* | EU vs. TE (p<0.01) EU vs. TL (p<0.01) EU vs. AE (p<0.01) EU vs. AL (p<0.05) TE vs. AL (p<0.05) TL vs. AE (p<0.05) TL vs. AL (p<0.01) |
